# An event-driven hybrid rescheduling approach for integrated process planning and scheduling considering stochastic rework

**DOI:** 10.1038/s41598-026-51261-z

**Published:** 2026-05-04

**Authors:** Shuangyuan Shi, Chang Liu, Lvjiang Yin, Hegen Xiong, Fang Xu, Chang Li, Ying Liu

**Affiliations:** 1 School of Computer and Information Science, Hubei Engineering University, Xiaogan, China; 2 Yangtze Delta Region Institute of Tsinghua University, Jiaxing, China; 3 School of Economics and Management, Hubei University of Automotive Technology, Shiyan, China; 4 School of Mechanical Engineering and Automation, Wuhan University of Science and Technology, Wuhan, China; 5 School of Mechanical Engineering, Hubei Engineering University, Xiaogan, China

**Keywords:** Integrated process planning and scheduling, Rework, Adaptive large neighborhood search, Reinforcement learning, Engineering, Mathematics and computing

## Abstract

While the static integrated process planning and scheduling (IPPS) problem is theoretically well-established, its practical application is limited in unpredictable manufacturing environments demanding dynamic adaptability. This paper proposes a dynamic IPPS problem considering stochastic rework (IPPS-SR), whose solution optimizes product quality and scheduling performance when imperfect items require reprocessing. We first formulate a mathematical optimization model for IPPS-SR that minimizes makespan and schedule instability, and then present an event-driven hybrid rescheduling approach featuring two key innovations: (1) a hybrid strategy that integrates right-shift scheduling with a multi-objective reinforcement learning-guided adaptive large neighborhood search (MORL-ALNS) algorithm, achieving an effective trade-off between computational efficiency and solution quality; and (2) a set of problem-specific operators, including five destroy and four repair operators, that enhance the search efficacy of the MORL-ALNS framework. Experimental results on 24 adapted benchmark instances indicate that the proposed hybrid approach effectively generates high-quality rescheduling schemes for the IPPS-SR problem. Specifically, the RL-guided mechanism increases the number of non-dominated solutions by over 80% on average compared to the baseline ALNS. Comprehensive experiments with other widely used multi-objective algorithms further demonstrate that MORL-ALNS achieves superior Hypervolume (HV) values in 22 out of 24 instances and lower Inverted Generational Distance (IGD) values in 23 out of 24 instances.

## Introduction

Process planning and scheduling are critical and interdependent functions in manufacturing industry. Process planning defines the process route and allocates resources, while scheduling arranges the sequence and timing of operations based on that plan. The conventional sequential execution of these activities in isolation restricts dynamic adjustment and overlooks valuable synergies between their often conflicting objectives^1–3^. To overcome these limitations, the integrated process planning and scheduling (IPPS) problem has been widely adopted in fields such as semiconductor manufacturing^4^, aerospace manufacturing^5^, and supply chain logistics^6^. However, research has predominantly focused on the static IPPS (SIPPS) problem due to its relatively simpler, deterministic nature, rather than the more complex but realistic dynamic IPPS (DIPPS) problem which handles real-time production disruptions.

**Fig. 1:**
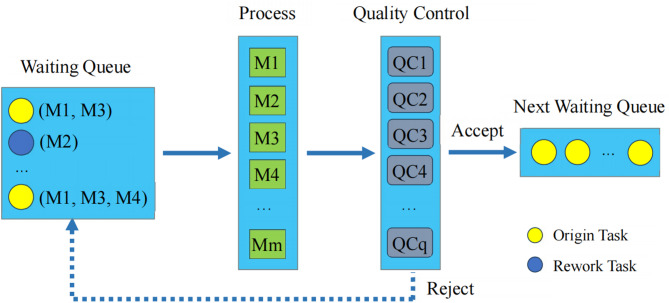
A manufacturing system with rework process configuration.

Among various dynamic disruptions, quality rework is a prevalent event that frequently disrupts initial schedules and increases overall production workload^7^. As illustrated in Fig. 1, when a job is identified as requiring rework following quality inspection, subsequent adjustments to the initial schedules are necessary to maintain system coordination and operational efficiency. Despite its real-world significance, existing research on this specific intersection remains relatively limited. To address this gap, our research aims to contribute by exploring this dynamic event.

Accordingly, this study considers quality rework as a key dynamic event and integrates it with the multiple flexible characteristics of IPPS problems (IPPS-SR). To ensure the stability of production in this dynamic environment, an event-driven hybrid rescheduling strategy based on a reinforcement learning (RL)-guided adaptive large neighborhood search heuristic (ALNS) is proposed. Two objectives are considered: makespan minimization and the minimization of schedule instability. The latter objective directly measures rescheduling stability, which is essential for mitigating production disruptions and improving operational efficiency and cost-effectiveness^1^. The main contributions of this paper are summarized as follows: (1) A multi-objective mathematical optimization model for IPPS-SR is formulated; (2) An event-driven hybrid rescheduling approach that effectively integrates complete rescheduling and right-shift scheduling is proposed to handle rework disruptions; (3) A RL-guided ALNS framework is designed, incorporating an object-oriented encoding strategy for efficient complete rescheduling. Five tailored destroy operators and four dedicated repair operators are designed to navigate the solution space; (4) Comprehensive experiments are conducted on 24 modified IPPS benchmark datasets, and the results demonstrate that the proposed approach effectively solves the IPPS-SR problem.

The remainder of this paper is organized as follows. Section  “Literature review” reviews the research work related to our topic. Section “Problem formulation” describes the proposed problem and establishes a mathematical model for it. The developed scheduling strategy and solving algorithm are introduced in section  “Solution approach”. Section  “Numerical experiments and discussion” presents numerical experiments and discussions. Finally, conclusions and suggestions for future research are summarized in section  “Conclusions and future works”.

## Literature review

### Review on DIPPS problems

IPPS problems are generally divided into two types: Type-Ⅰ (i.e., the process plans are enumerated in advance) and Type-II (i.e., the process plans are represented by AND/OR graphs). And three types of flexibility are considered in IPPS: processing flexibility (PF), operation flexibility (OF) and sequencing flexibility (SF). Existing studies on IPPS primarily focus on the scheduling strategies and solution algorithms for SIPPS problems. Zhu et al.^8^ constructed a constraint programming model for two types of IPPS problems, and an enhanced logic-based Benders decomposition (LBBD) algorithm was designed. For Type-II IPPS problems, Naderi et al.^9^ and Barzanji et al.^10^ developed approaches based on LBBD method, Zhang et al.^11^ presented a graph-based constraint programming approach. Cao and Shi^12^ proposed an adaptive multi-strategy artificial bee colony approach to address the SIPPS problem. Two search strategies with distinct characteristics were incorporated to enhance both exploration and exploitation. A nested approach integrating harmony search with genetic algorithm was introduced by Wu and Li^3^. This approach was divided into two parts, with the inner layer using the genetic algorithm and the outer layer using the harmony search algorithm. To enhance population diversity, the selection operator and minimum spanning tree clustering method were utilized. To address a green multi-objective IPPS problem minimizing makespan, tardiness, and carbon emissions, Wen et al.^13^ proposed a two-stage NSGA-II framework with three novel strategies for inter-stage information exchange. Ba et al.^14^ addressed a multi-objective just-in-time IPPS problem that minimizes machine workload (both total and maximum) and earliness/tardiness penalties. They developed a non-dominated sorting genetic algorithm featuring a self-adaptive decoding mechanism to solve it. Liu et al.^15^ designed a modified genetic algorithm based on the integrated encoding and decoding methods. In the context of distributed manufacturing, a mixed-integer linear programming model based on network was established by Liu et al.^16^. To address this challenge, a discrete artificial bee colony algorithm was designed.

Nowadays, the state of research on DIPPS is still in its infancy. The available literature on DIPPS problems is summarized in Table 1. Xia et al.^2^ formulated a DIPPS model considering the new job arrival and machine breakdown. The proposed model was solved using a hybrid genetic algorithm with variable neighborhood search. Wen et al.^1^ studied a DIPPS problem under machine fault. Two hybrid algorithms that combine a genetic algorithm and a neighborhood search algorithm were developed. In order to enhance the stability of the rescheduling result, a methodology for process adaptation founded upon job classification was presented. Jin et al.^17^ studied the characteristics of new job arrivals. A novel mixed integer linear programming model was formulated, and three objectives were considered: stability, tardiness, and makespan. Erden et al.^18^ developed four metaheuristics for the dynamic IPPS problem with due-date assignment (DIPPSDDA), which considers new job arrivals, to minimize earliness and tardiness. For uncertain characteristics in real-world manufacturing environments, a multi-objective IPPS problem with fuzzy due date and uncertain processing time was tackled by Wen et al.^19^. And the problem was solved by an effective modified honey bees mating optimization algorithm. While the literature on DIPPS has explored various dynamic events, research specifically addressing the IPPS-SR problem remains scarce. To the best of our knowledge, the integration of stochastic rework into the IPPS framework has not yet been scientifically investigated.

As shown in Table 1, existing research has explored the DIPPS problem with the arrival of new jobs. Although the arrival of new jobs and the rework problem may appear similar superficially, they are inherently distinct. Rework represents a local disruption within the production system, while the arrival of new jobs constitutes a global integration from external sources. Rework pertains to already scheduled tasks, with its core objective being localized adjustments and revisions via PF, OF, and SF to minimize deviations from the original scheduling plan. In contrast, the arrival of new jobs fundamentally alters the composition of the task set, requiring global resource reallocation and full rescheduling of the entire system. Accordingly, the incorporation of job rework into the IPPS problem also bears considerable academic value and practical significance.

**Table 1:** Review of studies on dynamic events in IPPS problems.

Reference	Dynamic event				Solution approach	Objective function
	NJI	MB	OC	QR		
Xia et al.^2^	√	√			Genetic algorithm + Variable neighborhood search	Makespan Mean flow time
Wen et al.^1^		√			Genetic algorithm/NSGA-II + variable neighborhood search	Makespan Operation deviation Machine deviation
Jin et al.^17^	√				NSGA-II	Makespan Stability Tardiness
Wong et al.^20^	√	√			Online hybrid agent-based negotiation	Flowtime Machine utilization Deviations from the preschedule
Lv and Qiao^21^	√	√	√		Improved evolutionary algorithm + Machine adjusting algorithm	Makespan Mean flow time
Yin et al.^22^	√	√			Genetic algorithm	Makespan Mean flow time
Guo et al.^23^	√	√			Particle swarm optimization algorithm	Makespan Tardiness Machine utilization
Erden et al.^18^	√				Genetic algorithm + tabu algorithm + simulated annealing + hybrid algorithms	Earliness and tardiness
Our work				√	Adaptive large neighborhood search heuristic	Makespan Deviations from the preschedule

Notes: NJI (new job insertion); MB (machine breakdown); OC (order cancellation); QR (quality rework).

### Rework process in manufacturing systems

In the majority of real-world manufacturing systems, it is an inherent consequence of the production process that some items of inferior quality will be produced. Some defective items are produced unexpectedly and require reworking or disposal. However, for expensive products or assemblies, such as injection molding, semiconductor and printed circuit board assemblies, companies usually opt to perform rework processes to transform defective items into serviceable ones rather than scrap them^24^. Hence, the rework process can reduce waste and affect production costs. It can also have an impact on the original manufacturing schedule. Therefore, it is valuable to investigate the optimal timing for implementing a defective rework process within an imperfect production system.

Numerous studies have explored the effects of rework on the production process^24–26^. Sha et al.^27^ developed a set of operative dispatching rules in the photolithography area of wafer fabrication while taking the rework process into consideration. The present study concentrated on the batch with high finished proportion to identify solutions to increase production output, reduce the machine workload in waiting queue, and complete the manufacturing process faster. A production system with an imperfect reworking process and a Poisson machine breakdown rate was addressed by Chiu et al.^28^. Liu and Zhou^29^ addressed an issue on identical parallel machines where the original jobs were arranged to minimize the job rework disruption, and the total completion time was considered. Two conflicting rescheduling criteria were designed. Rabiee et al.^30^ addressed a no-wait two-stage flexible flow shop scheduling problem with different ready times, probable reworks, sequence-dependent setup times, and unrelated parallel machines consideration. A novel intelligent hybrid algorithm combining simulated annealing, genetic algorithm, and variable neighborhood search was developed to solve the proposed problem. Shin^31^ presented a parallel machine scheduling problem with sequence-dependent setup time, due dates, and process quality. A new dispatching algorithm was proposed concerning the rework process due to quality. Quan et al.^32^ considered a multi-objective optimization scheduling problem considering key operation reworking. A multi-objective evolutionary scheduling algorithm with adaptive rules and a two-level virtual workflow modeling method was designed for solving the problem.

Consequently, it is imperative to develop effective strategies for the rework process to simultaneously enhance production quality and scheduling performance. However, the IPPS problem that incorporates rework operations remains unaddressed in existing research.

### Dynamic scheduling methods

Achieving a stable scheduling scheme under dynamic environments remains a major challenge. Existing approaches for solving dynamic scheduling problems can be broadly categorized into three strategies: completely reactive scheduling, pre-reactive scheduling and robust scheduling^33,34^. Among these, pre-reactive scheduling is widely adopted in practical applications and serves as the methodological foundation for this study. Under the pre-reactive scheduling framework, two key decisions are involved in the rescheduling process: rescheduling moment (i.e., determining when to trigger rescheduling) and rescheduling method (i.e., defining how to execute rescheduling)^35,36^. In general, the primary methods for identifying rescheduling moments include event-driven rescheduling, periodic rescheduling, and hybrid rescheduling; and the main rescheduling methods include complete rescheduling, partial rescheduling, and right-shift scheduling^1^.

In real manufacturing environments, production is typically a highly dynamic process, fraught with various unexpected events. Consequently, workshop managers must consider a range of strategies, policies, and approaches to effectively navigate these ever-changing environments. Zhu et al.^37^ studied a distributed flexible job shop scheduling problem (FJSSP) involving operation inspection. For the initial scheduling scheme, a modified memetic algorithm was designed for minimizing the total energy consumption and makespan. During the rescheduling stage, the event-driven predictive reactive scheduling strategy was utilized, a hybrid rescheduling method that incorporates three rescheduling strategies was designed to manage diverse inspection results. Liu et al.^38^ and Rahmani and Heydari^39^ proposed a proactive-reactive approach to address dynamic events, including unexpected arrivals of new jobs, uncertain processing times, and stochastic machine breakdown. The approach was comprised of two distinct steps. In the initial step, a robust solution was developed. In the subsequent step, an effective rescheduling approach was proposed. Liu and Urgo^40^ dealt with a two-machine re-entrant flow shop scheduling problem considering stochastic processing times, in which each job needs a rework phase. To devise robust schedules aimed at minimizing the value-at-risk associated with the makespan, a branch-and-bound algorithm along with heuristic algorithms were proposed.

For a detailed explanation of the dynamic scheduling method, please refer to Ouelhadj and Petrovic^34^ and Wen et al.^1^. Pre-reactive scheduling is a dynamic scheduling approach commonly used in shop scheduling, which involves analyzing and adjusting the scheduling scheme in response to unexpected disruptions in the shop floor production environment. This methodology is also applied in this study to investigate IPPS-SR.

### Adaptive large neighborhood search heuristic

Adaptive large neighborhood search (ALNS) has recently gained interest and achieved success. This method originated from the large neighborhood search introduced by Shaw^41^. The ALNS framework features a dual-loop structure: an inner loop performing local search with destroy and repair operators, and an outer loop guiding the overall process through acceptance criteria. This structure enables extensive neighborhood reconstruction and powerful global search, making it highly effective for discrete combinatorial optimization problems^42,43^. Notably, it has been successfully applied to a wide range of vehicle routing problems and production scheduling problems^44–46^.

Mara et al.^42^ provided a survey on the variant of ALNS features, application areas, and the publication intensity on 252 scientific publications. Rifai et al.^47^ devised a new multi-objective ALNS algorithm that can simultaneously minimize average tardiness, total cost, and makespan for distributed permutation flow shop scheduling problems. To balance the search process’s intensification and diversification, six destroy operators and four repair operators were developed. Fang et al.^48^ and Rolim et al.^49^ tackled an unrelated parallel machine scheduling problem. Several removal/insertion operators were integrated into an ALNS framework. Ji et al.^50^ investigated a novel FJSSP considering the machines’ batch-processing capabilities. Tabu-based components and an optimal repair operator were proposed for ALNS. In order to improve the exploitation and exploration ability of the algorithm, a perturbation strategy was designed. The preceding literature review has demonstrated that the ALNS algorithm has the capacity to yield promising outcomes in production scheduling problems. The inherent flexibility of the ALNS framework allows us to tailor the destroy and repair operators to the specific characteristics of the dynamic scheduling problems. Nevertheless, the ALNS algorithm has yet to be studied for solving IPPS problems, and there is still scope for further improvements to ALNS for specific multi-objective optimization problems.

In summary, it is necessary to investigate the IPPS problem to ensure the production quality when defective items are produced unexpectedly. An event-driven predictive-reactive scheduling method has been developed for Type-II IPPS problems, taking into account the rework process. The ALNS framework, equipped with five destroy operators and four repair operators, is utilized. The designed method for obtaining the initial scheduling scheme has been evaluated on 24 IPPS benchmarks. The impact of multiple flexibilities and the stability of the proposed MORL-ALNS have been verified on modified benchmarks, and a comparison of MORL-ALNS with other well-known multi-objective algorithms is presented for tackling the IPPS-SR problem.

## Problem formulation

In this section, the proposed problem of IPPS-SR is described, and a multi-objective optimization mathematical model is formulated.

### Problem description

The problem of IPPS-SR can be described as follows. There is a set of *n* independent jobs, denoted as ${\boldsymbol{J}}=\left\{ {{J_1},{J_2}, \ldots ,{J_n}} \right\}$, which need to be processed on a set of *m* machines, denoted as ${\boldsymbol{M}}=\left\{ {{M_1},{M_2}, \ldots ,{M_m}} \right\}$. Three types of flexibility (i.e., PF, OF and SF) are considered in this problem. Each job has a varying number of processing features, and there are sequential constraints among these features. Each feature can be completed by one or more available processing routes, each consisting of a different number of operations. Each operation must be processed by one of its candidate machines, and its processing time is machine-dependent. If an operation is processed imperfectly, it must be reworked or remanufactured, and such situations may occur randomly. In this study, the occurrence of rework is modeled as a stochastic event, where each operation has a predefined rework probability $rp$ that reflects the inherent quality uncertainty in production.

The primary purpose of this study is to develop a method that (1) selects an appropriate processing route for each job, (2) assigns a suitable machine to each operation, and (3) determines the starting time of each operation. If an operation requires rework during the production, a rescheduling procedure is triggered. The objective functions of this research are to minimize the maximum completion time of all jobs (i.e., makespan) and to minimize the deviation between the initial scheduling scheme and the rescheduling scheme.

The following assumptions are made in IPPS-SR.

1. All machines and jobs are available at the beginning.
2. A machine can process only one job at a time, and each job can be processed by only one machine at a time.
3. The time of setup and job transportation is not considered separately.
4. The rework time equals the original processing time if the rework machine is identical to the original processing machine.
5. Machine fault is ignored.
6. The sequential order between features is predefined in advance.

**Fig. 2:**
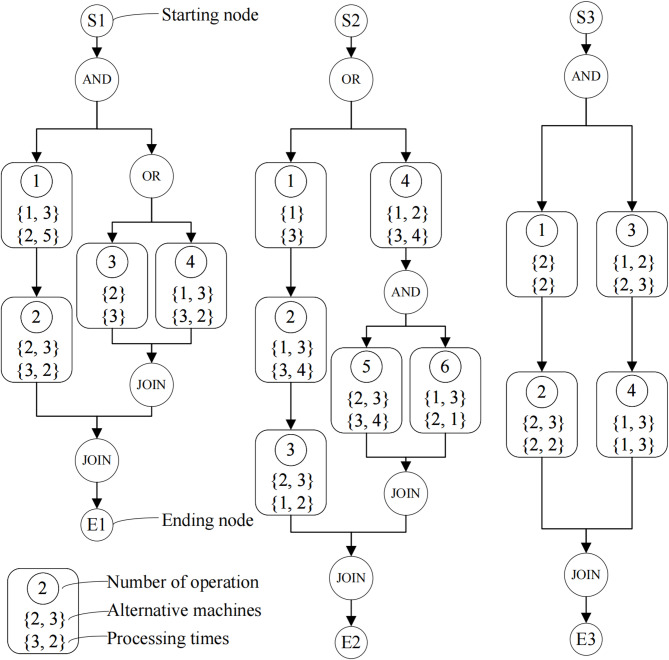
The network graphs of three sample jobs.

To represent an IPPS-SR problem, the AND/OR graph is an effective and clear method for describing the complete set of feasibilities^51^. Figure 2 depicts the alternative process routes, sequence relationships, and relevant operations for three sample jobs. There are six node types in the graph: starting node, ending node, AND node, OR node, JOIN node, and operation node. The starting node (e.g., S1, S2, and S3) and ending node (e.g., E1, E2, and E3) are dummy ones, and indicate the start and the completion of a job, respectively. AND nodes are employed to represent sequential substitutability, wherein the operation sequences across distinct AND-link (the links connected by an AND node are called AND-links) paths can be interchanged while maintaining the precedence constraints within each individual AND-link path. OR nodes are utilized to represent operational substitutability, where only a single path among the available OR-links requires traversal. The termination of the AND/OR-link path is marked by a JOIN node. An operation node encapsulates a specific manufacturing process, including the alternative machines and corresponding processing time required for the operation.

**Fig. 3:**
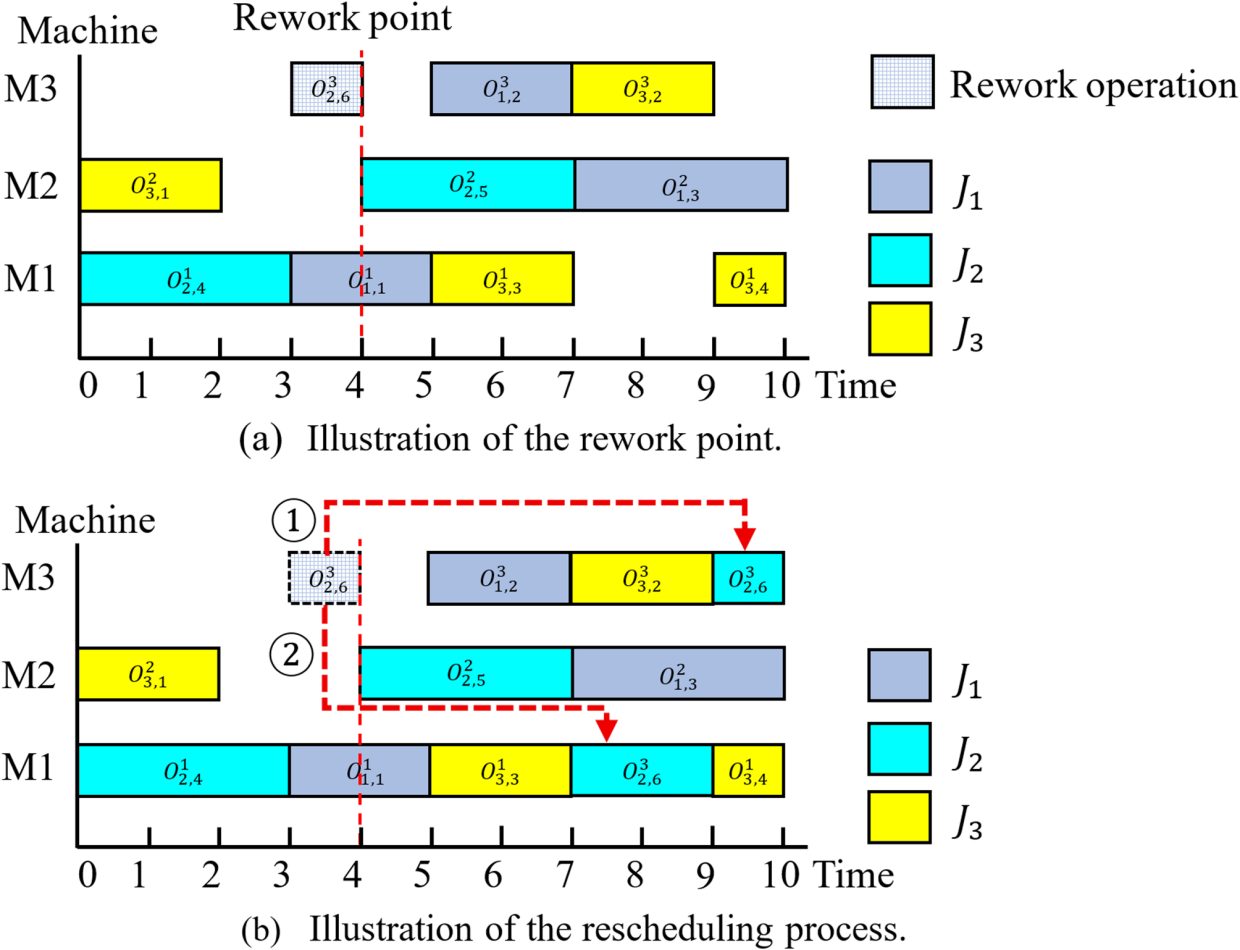
Example of the rework point and the rescheduling process.

The feasible process plans for these three jobs in Fig. 2 are shown in Table 2. Job 1, 2, and 3 have 4, 3, and 4 feasible process plans, respectively. Figure 3 displays an initial scheduling result for job 1, job 2 and job 3. Suppose that operation $O_{{2,6}}^{3}$ requires rework after the quality inspection, it is necessary to determine whether a rescheduling scheme needs to be triggered. At the rework point, the operations can be categorized into three types: the set of finished operations (i.e., $O_{{2,4}}^{1}$ and $O_{{3,1}}^{2}$), the set of being processed operations (i.e., $O_{{1,1}}^{1}$), and the set of operations to be processed (i.e., $O_{{2,5}}^{2}$, $O_{{1,2}}^{3}$, $O_{{1,3}}^{2}$, $O_{{3,2}}^{3}$, $O_{{3,3}}^{1}$ and $O_{{3,4}}^{1}$). During the rescheduling stage, the set of finished operations should be excluded, and the three flexibilities (i.e., PF, SF and OF) should be fully considered to generate new process routes and scheduling schemes for obtaining better production plans. It can be seen from Fig. 3 that although the operation $O_{{2,6}}^{3}$ requires rework, as $O_{{2,5}}^{2}$ and $O_{{2,6}}^{3}$ are within the same AND-link path, the processing of $O_{{2,6}}^{3}$ can be rescheduled to follow $O_{{3,2}}^{3}$ (processing time remains 1) or $O_{{3,3}}^{1}$ (processing time is 2) without affecting the makespan or modifying the start times of other operations (see Fig. 3(b)). In response to process rework scenarios, differentiated rescheduling strategies should be implemented based on the real-time workshop processing status, aiming to minimize disruptions to the initial scheduling scheme while ensuring the stability of production plans and operational efficiency. As can be seen from Fig. 3, although rework was performed on operation $O_{{2,6}}^{3}$, the rescheduling strategy ensured that the starting and completion times of other operations are not affected.

**Table 2:** Feasible process plans for three sample jobs.

Job index	Feasible process plans
Job 1	${O_{1,1}} - {O_{1,2}} - {O_{1,3}},{O_{1,1}} - {O_{1,2}} - {O_{1,4}},{O_{1,3}} - {O_{1,1}} - {O_{1,2}},{O_{1,4}} - {O_{1,1}} - {O_{1,2}}$
Job 2	${O_{2,1}} - {O_{2,2}} - {O_{2,3}},{O_{2,4}} - {O_{2,5}} - {O_{2,6}},{O_{2,4}} - {O_{2,6}} - {O_{2,5}}$
Job 3	${O_{3,1}} - {O_{3,2}} - {O_{3,3}} - {O_{3,4}},{O_{3,1}} - {O_{3,3}} - {O_{3,2}} - {O_{3,4}},{O_{3,3}} - {O_{3,1}} - {O_{3,2}} - {O_{3,4}},{O_{3,3}} - {O_{3,1}} - {O_{3,4}} - {O_{3,2}}$

### Mathematical model for IPPS-SR

The notations used to explain the IPPS-SR model are listed in Table 3.

**Table 3:** Notations in IPPS-SR mathematical model.

Symbol	Description
*n*	The number of jobs
*m*	The number of machines
$i,h$	Index of jobs, $i,h=1,2, \ldots ,n$
*k*	Index of machines, $k=1,2, \ldots ,m$
${{\boldsymbol{O}}_i}$	The set of all operation for job *i*
$j,l$	Index of operations
${O_{i,j}}$	The $j{\mathrm{th}}$ operation of job *i*
$O_{{i,j}}^{k}$	The $j{\mathrm{th}}$ operation of job *i* is processed by machine *k*
$n_{i}^{{total}}$	The total operation number of job *i*
${n_i}$	The actual operation number of job *i* after its process plan is determined
${{\boldsymbol{M}}_{i,j}}$	The set of candidate machines for operation ${O_{i,j}}$
$\left| {{{\boldsymbol{M}}_{i,j}}} \right|$	The number of candidate machines for operation ${O_{i,j}}$
${P_{i,j,k}}$	The processing time of ${O_{i,j}}$ on machine *k*
$P_{{i,j,k}}^{\prime }$	The rework processing time of ${O_{i,j}}$ on machine *k*.
*A*	An infinite positive number
${C_i}$	The completion time of job *i*
${C_{max}}$	The maximum completion time of all jobs
${S_{i,j}}$	The starting time of operation ${O_{i,j}}$ in the initial scheduling scheme
${S_{i,j,k}}$	The starting time of operation ${O_{i,j}}$ on machine *k* in the initial scheduling scheme
$S_{{i,j}}^{\prime }$	The starting time of operation ${O_{i,j}}$ in the rescheduling scheme
${C_{i,j}}$	The completion time of operation ${O_{i,j}}$
${C_{i,j,k}}$	The completion time of operation ${O_{i,j}}$ on machine *k*
$rp$	The rework probability of all operations
${Q_{i,j}}$	A binary value. If the $j{\mathrm{th}}$ operation of job *i* is selected in the initial scheduling scheme, ${Q_{i,j}}=1$; otherwise ${Q_{i,j}}=0$
$Q_{{i,j}}^{\prime }$	A binary value. If the $j{\mathrm{th}}$ operation of job *i* is selected in the rescheduling scheme, $Q_{{i,j}}^{\prime }=1$; otherwise $Q_{{i,j}}^{\prime }=0$
${R_{i,j}}$	A binary value. If the $j{\mathrm{th}}$ operation of job *i* is a rework operation, ${R_{i,j}}=1$; otherwise ${R_{i,j}}=0$
${X_{i,j,k}}$	A binary value. If operation ${O_{i,j}}~$is assigned to machine *k* in the initial scheduling scheme, ${X_{i,j,k}}=1$; otherwise ${X_{i,j,k}}=0$
$X_{{i,j,k}}^{\prime }$	A binary value. If operation ${O_{i,j}}~$is assigned to machine *k* in the rescheduling scheme, $X_{{i,j,k}}^{\prime }=1$; otherwise $X_{{i,j,k}}^{\prime }=0$
${Y_i}\left( {j,l} \right)$	A binary value. If operation ${O_{i,j}}~$is processed before operation ${O_{i,l}}$, ${Y_i}\left( {j,l} \right)=1$; otherwise ${Y_i}\left( {j,l} \right)=0$
${Z_{i,j,h,l,k}}$	A binary value. If operation ${O_{i,j}}~$is processed before operation ${O_{h,l}}$ on machine *k*, ${Z_{i,j,h,l,k}}=1$; otherwise ${Z_{i,j,h,l,k}}=0$

The optimization model can be formulated as follows:

Objective:

1. Minimize makespan

1 $$f1=\hbox{min} \left( {{C_{max}}} \right)=\hbox{min} \left( {\hbox{max} \left\{ {{C_i}} \right\}} \right),~~~~i=1,2, \ldots ,N$$

Makespan refers to the maximal completion time for all jobs.

(2) Minimize operation and machine selection deviation (*OMD*)

2 $$f2=\hbox{min} \left( {OMD} \right)=min\mathop \sum \limits_{{i=1}}^{n} \mathop \sum \limits_{{j=1}}^{{{n_i}}} \left( {\left| {{Q_{i,j}} - Q_{{i,j}}^{\prime }} \right|+\mathop \sum \limits_{{k=1}}^{{\left| {{{\boldsymbol{M}}_{i,j}}} \right|}} \left| {{Q_{i,j}}Q_{{i,j}}^{\prime }\left( {{X_{i,j,k}} - X_{{i,j,k}}^{\prime }} \right)} \right|/2} \right)$$

*OMD* refers to the sum of the number of operation and machine selection deviations before and after the rework process. A smaller *OMD* value indicates less disruption to the workshop production.

*s.t.*

3 $${S_{i,j}} \geqslant 0,~~~~i=1,2, \ldots ,N;j=1,2, \ldots ,n_{i}^{{total}}$$

4 $${S_{i,j}}+{P_{i,j,k}}+P_{{i,j,k}}^{\prime } \leqslant {S_{i,j+1}},~~~~i=1,2, \ldots ,n;j=1,2, \ldots ,n_{i}^{{total}}$$

5 $$P_{{i,j,k}}^{\prime }={R_{i,j}}{P_{i,j,k}}$$

6 $$\mathop \sum \limits_{{k=1}}^{m} {X_{i,j,k}}=1,~~~~~~i=1,2, \ldots ,n;j=1,2, \ldots ,n_{i}^{{total}}$$

$${C_{i,j}}={S_{i,j}}+{Q_{i,j}}{X_{i,j,k}}\left( {{P_{i,j,k}}+P_{{i,j,k}}^{\prime }} \right),~~~~~i=1,2, \ldots ,n;$$

7 $$~j=1,2, \ldots ,n_{i}^{{total}};k=1,2, \ldots ,\left| {{{\boldsymbol{M}}_{i,j}}} \right|$$

8 $${C_{i,j}} \leqslant {S_{i,l}}+A\left( {1 - {Y_i}\left( {j,l} \right)} \right),~~~~i=1,2, \ldots ,n;j,l=1,2, \ldots ,n_{i}^{{total}}$$

$${S_{i,j,k}}+{P_{i,j,k}} \leqslant {S_{h,l,k}}+A\left( {1 - {z_{i,j,h,l,k}}} \right)~~$$

9 $$i,h=1,~2,~ \ldots ,~n,~~~j,l=1,2, \ldots ,{n_i},~k=1,2, \ldots ,\left| {{{\boldsymbol{M}}_{i,j}}} \right|$$

10 $${Q_{i,j}},~Q_{{i,j}}^{\prime } \in \left\{ {0,1} \right\},~~~~i=1,2, \ldots ,n;j=1,2, \ldots ,n_{i}^{{total}}$$

11 $${X_{i,j,k}}~X_{{i,j,k}}^{\prime } \in \left\{ {0,1} \right\},~~~~i=1,2, \ldots ,n;j=1,2, \ldots ,n_{i}^{{total}};k=1,2, \ldots ,\left| {{{\boldsymbol{M}}_{i,j}}} \right|$$

12 $${Y_i}\left( {j,l} \right) \in \left\{ {0,1} \right\},~~~~i=1,2, \ldots ,n;j,l=1,2, \ldots ,n_{i}^{{total}}$$

13 $${z_{i,j,h,l,k}} \in \left\{ {0,1} \right\},~~~~i,h=1,2, \ldots ,n;j,l=1,2, \ldots ,n_{i}^{{total}};k=1,2, \ldots ,\left| {{{\boldsymbol{M}}_{i,j}}} \right|$$

Equations (1) and (2) denote the objective functions: minimizing the makespan and the deviation between the initial scheduling scheme and the rescheduling scheme. The deviation described in Eq. (2) consists of two parts: the first part represents the operation deviation, and the second part indicates the machine deviation. Constraint (3) and (4) guarantee the starting time of an operation and the precedence relationship between two operations within a job. Constraint (5) defines the rework processing time for a rework operation. Constraint (6) specifies that an operation should be processed on one of the alternative machines. Constraint (7) ensures that all operations must be completed without interruption once they start. Constraint (8) guarantees that each job can be processed on only one machine at a time. Constraint (9) ensures that only one operation can be processed on a given machine at any time. Constraint (10) to (13) define the binary variables.

## Solution approach

Given that solution methods for the static IPPS (SIPPS) problem are now well-established, this study is dedicated to the rescheduling phase of IPPS-SR. The initial solution for the problem is obtained following the method presented by Zhu et al.^8^ and Wen et al.^1^. To tackle the dynamic nature and multiple flexibilities involved in the IPPS-SR problem, this study introduces an event-driven hybrid rescheduling approach for the rescheduling phase.

### Solution framework

**Fig. 4:**
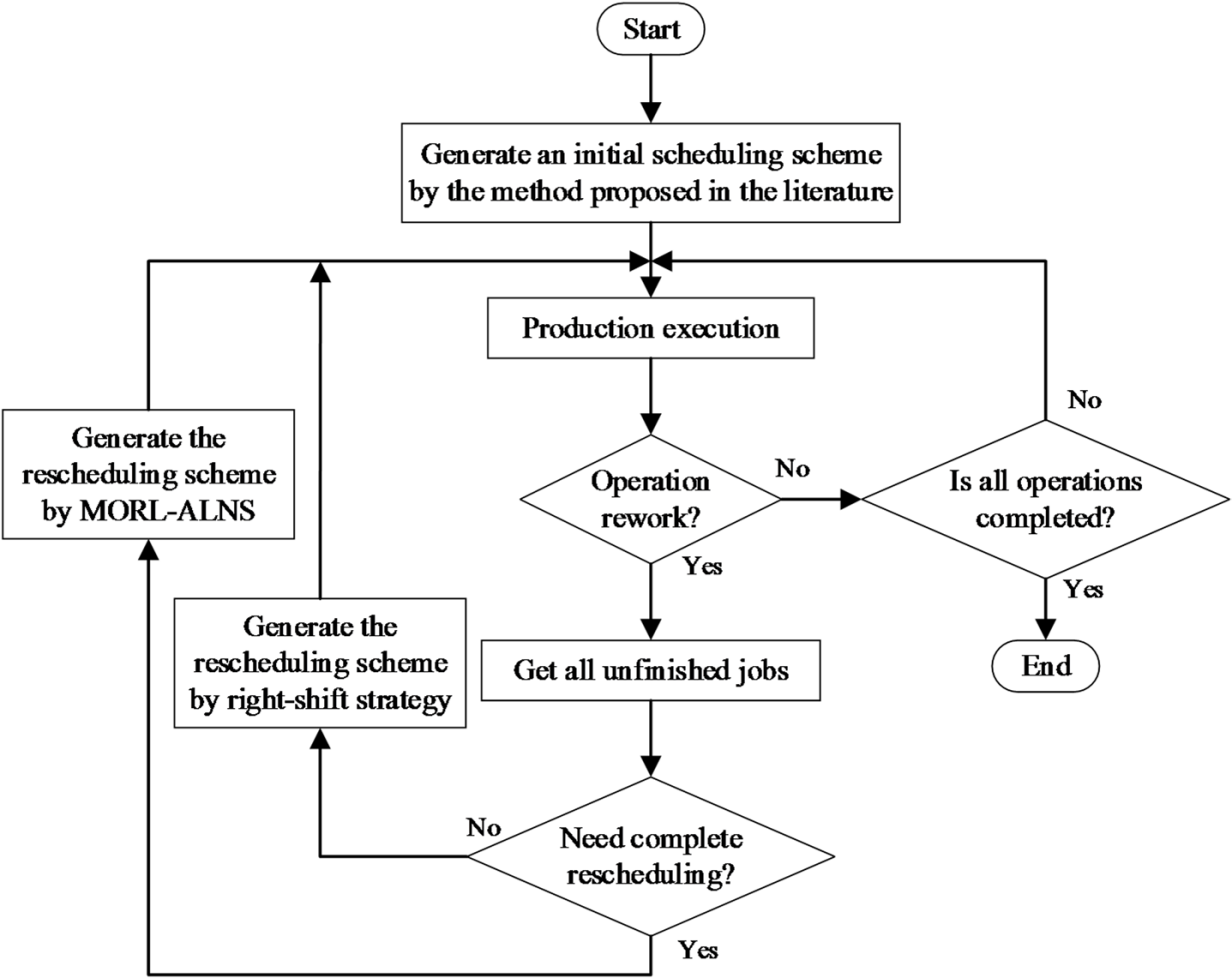
Event-driven rescheduling framework for IPPS-SR.

If an operation fails to meet quality standards and requires rework, the event-driven hybrid rescheduling approach is activated. This hybrid strategy integrates right-shift scheduling with complete rescheduling, achieving an effective trade-off between computational efficiency and solution quality by dynamically selecting between the two based on real-time shop-floor status. Specifically, right-shift scheduling simply shifts rework operations rightward along the timeline, a lightweight adjustment that minimizes disruption and preserves schedule stability for simple, isolated rework events. In contrast, complete rescheduling reprocesses all unfinished operations to handle complex rework scenarios where interdependent operations or resource conflicts necessitate global optimization. For complete rescheduling, a RL-guided ALNS framework is tailored with five destroy and four repair operators, each designed to exploit the three types of flexibility inherent in the IPPS-SR problem. These operators work synergistically to balance exploration and exploitation, enhancing search efficacy. The solving framework is illustrated in Fig. 4.

#### Framework for the right-shift strategy

**Fig. 5:**
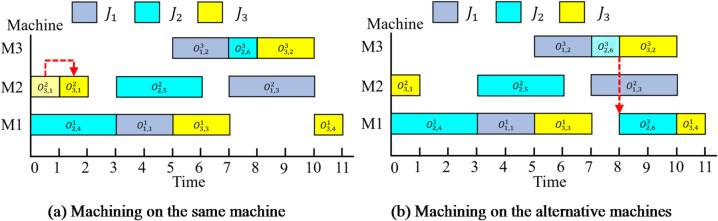
Scenarios that the right-shift scheduling strategy is used when the operation rework occurs. (a) Illustrates that the rework operation can be reprocessed on the same machine without delaying other operations. (b) Represents that the rework operation necessitates relocation to an alternative machine, due to insufficient idle time on the original machine.

The right-shift strategy is a rescheduling approach that aims to accommodate rework operations while preserving the original processing sequence and start times of the remaining unfinished operations as much as possible. Specifically, when a rework operation occurs, this strategy attempts to insert it into available idle time slots without delaying other operations. In the proposed framework, as illustrated in Fig. 4, it becomes essential to select an appropriate rescheduling method, namely complete rescheduling or right-shift scheduling, to perform the rescheduling procedure. Under the premise of ensuring the processing sequence and starting time of unfinished operations remain unchanged, and while satisfying the constraint relationships between operations of the same job, the feasibility of right-shift scheduling is evaluated based on two scenarios, as illustrated in Fig. 5: (1) Fig. 5(a) indicates that if the idle time on the original processing machine meets the processing requirements for the rework operation, the rework operation should be right-shifted to that time period for processing; (2) Fig. 5(b) indicates that if the condition of Fig. 5(a) is not met, and if the alternative machines for the rework operation meet the processing requirements, the rework operation can be directly right-shifted to one of those machines for processing. If there are multiple machines that meet the requirements, choose the one with the smallest processing time.

#### Framework for MORL-ALNS

**Fig. 6:**
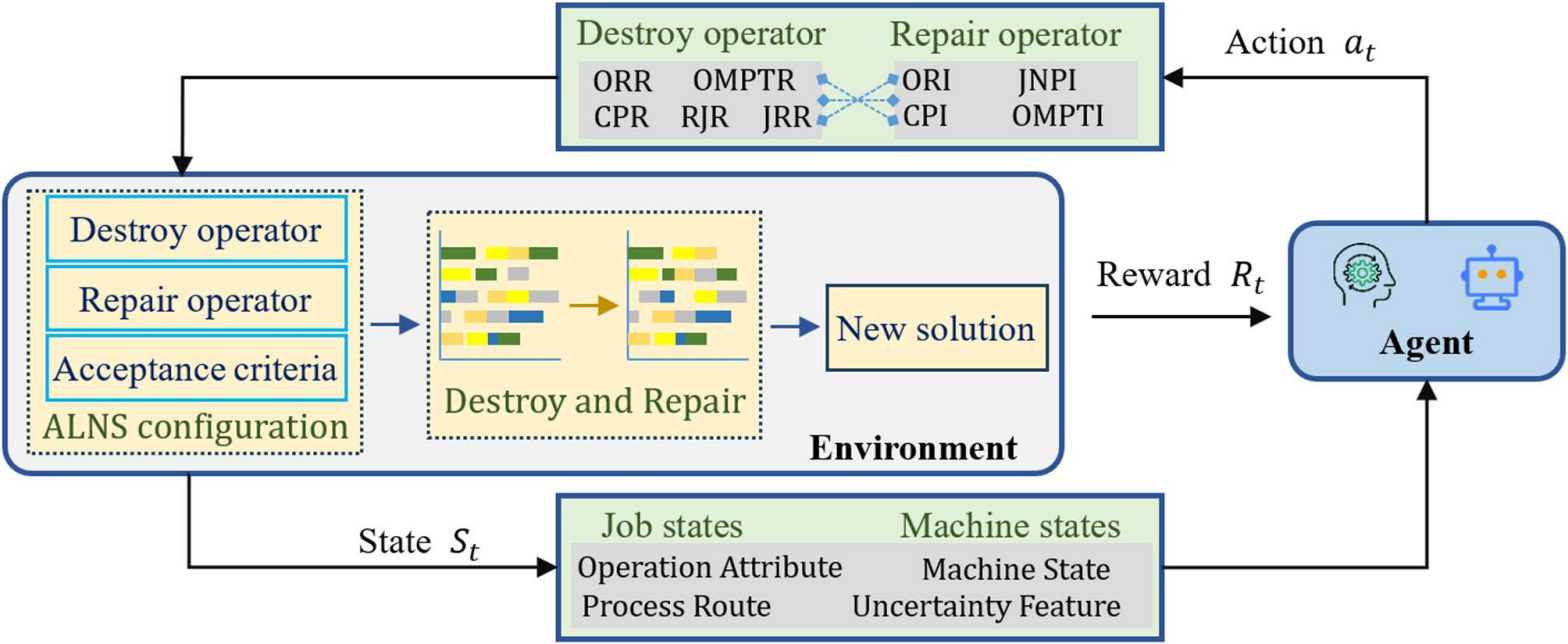
Framework of MORL-ALNS algorithm.

If neither of the scenarios in Fig. 5 is met, the MORL-ALNS algorithm is utilized to perform a complete rescheduling process for all unfinished operations. It is important to note that during the rescheduling phase, not all machines are immediately available because some operations on those machines are not yet completed at the time of rescheduling (referred to as the set of being-processed operations). Therefore, it is imperative to initialize this processing information at the parameter configuration stage. Additionally, an external Pareto archive set has been established to record the non-dominated solutions obtained during the iterations. The basic ALNS heuristic relies on a set of destroy operators (${\boldsymbol{\varOmega}^d}=\Omega _{1}^{d}, \ldots ,\Omega _{{\left| {{\boldsymbol{\varOmega}^d}} \right|}}^{d}$) and a set of repair operators (${\boldsymbol{\varOmega}^r}=\Omega _{1}^{r}, \ldots ,\Omega _{{\left| {{\boldsymbol{\varOmega}^r}} \right|}}^{r}$), and the framework of MORL-ALNS is presented in Fig. 6.

### Procedures in ALNS

The proposed ALNS procedure explores the solution space through repeated cycles of destruction and repair, guided by a RL mechanism. The following details the key components of this process.

#### Solution representation

1. Encoding scheme

**Fig. 7:**
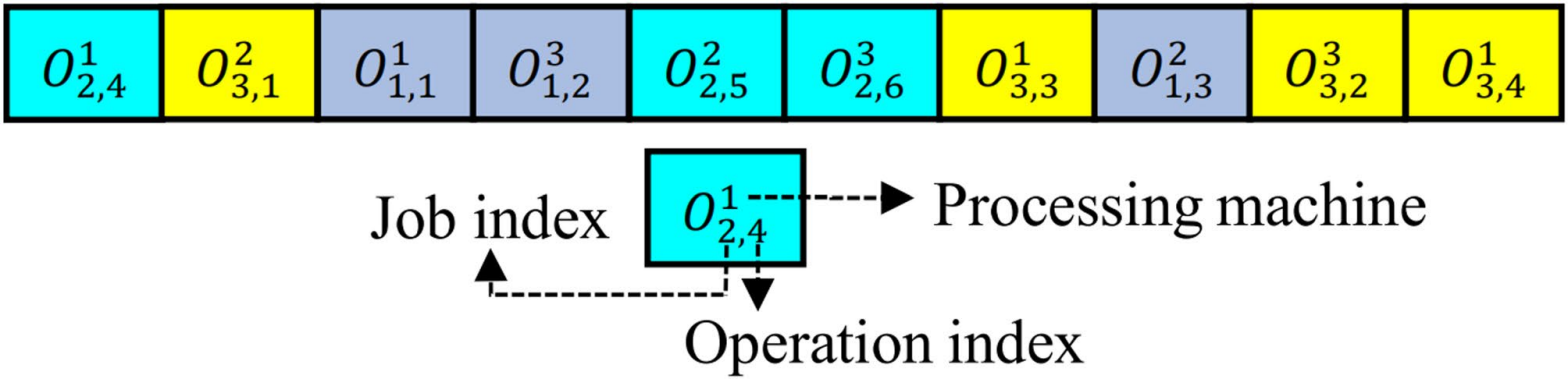
A sample chromosome for the sample IPPS problem in Table 2.

Inspired by the object-coding representation proposed by Zhang and Wong^52^, this study utilized the object-oriented encoding scheme (OOE) to construct solutions. The term “object” generally designates practical components, such as machines, operations, and jobs, etc. Specifically, in this study, an “object” denotes an operation performed on a dedicated processing machine. This representation requires only one chromosome to encapsulate all the information about an individual solution, and it reveals significantly more information that is directly accessible during the heuristic search process. Figure 7 presents a sample chromosome for the DIPPS problem described in Table 2. The locations of any two operations on a chromosome represent their precedence relationship.

(2) Decoding scheme

For the fitness evaluation, each chromosome representing an IPPS-SR solution can be decoded into several types of schedules based on the idle time between operations, including active schedules, semi-active schedules, and non-delay schedules^53,54^. In this study, when obtaining the initial scheduling solution, the chromosomes are decoded into active schedules because makespan is the optimization objective. To avoid falling into a local optimum and ensure the population diversity, in the rescheduling phase, 80% of the chromosomes are decoded into active schedules, while the remaining 20% are decoded into semi-active schedules.

**Fig. 8:**
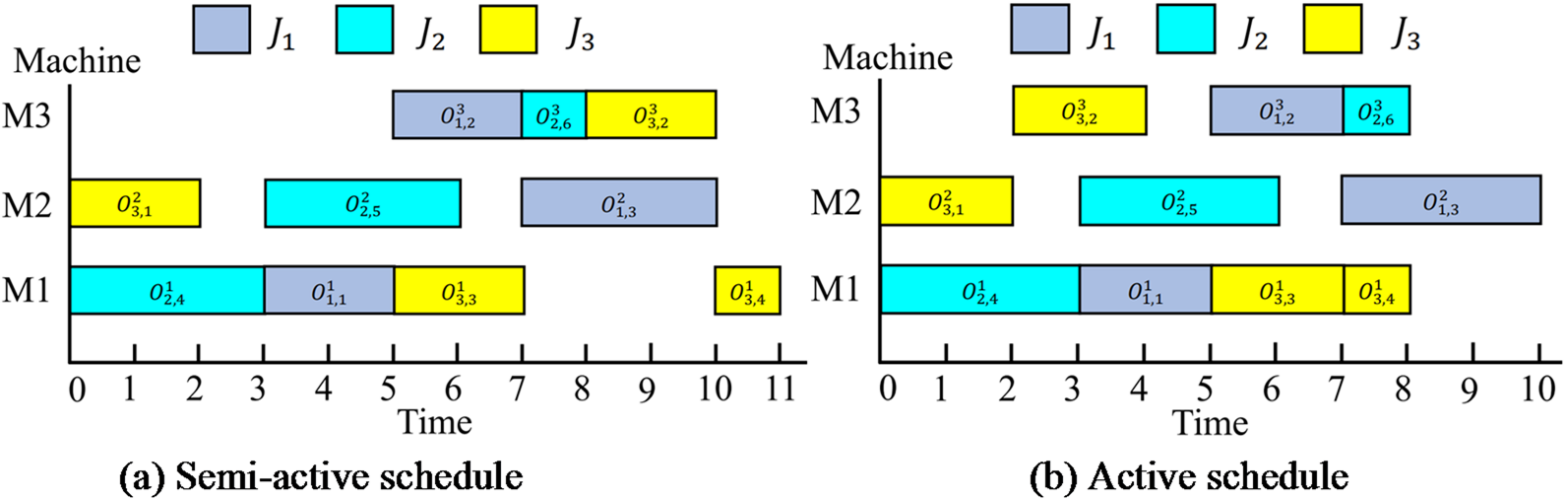
The Gantt chart of the decoding result for the sample chromosome illustrated in Fig. 7. For a semi-active schedule, no operation can be started earlier without changing the processing order on any of the machines. And for an active schedule, no operation can be started earlier without delaying other jobs. (a) Semi-active schedule, (b) Active schedule.

Figure 8 displays the semi-active schedule and the active schedule for the sample chromosome illustrated in Fig. 7. When decoding the sample chromosome into the semi-active schedule, the makespan is 11, as can be seen from Fig. 8 (a). To improve the makespan, a semi-active schedule can be converted to an active schedule by moving an operation to the left without causing any delay to other jobs^55^.

Take the semi-active schedule in Fig. 8 (a) as an example. When decoding $O_{{3,2}}^{3}$, idle time exists on $M3$ between $\left[ {0,{S_{1,2,3}}} \right]$, and ${S_{1,2,3}} - \hbox{max} \left\{ {0,{C_{3,1,2}}} \right\}>{P_{3,2,3}}$. Moreover, no sequential constraint exists between $O_{{3,2}}^{3}$ and $O_{{3,3}}^{1}$ due to sequence flexibility. Thus, the starting time of $O_{{3,2}}^{3}$ can be advanced to ${S_{3,2,3}}={C_{3,1,2}}$. The Gantt chart of the active schedule is shown in Fig. 8 (b). It can be observed that the makespan for the active schedule is 10, which is smaller than that of the semi-active schedule.

#### Destroy operators

The destroy operation removes certain elements from the current solution (${S_{current}}$) according to the destroy strategy, yielding a destroyed solution (${S_{destroyed}}$) with some constraints temporarily ignored. The removed elements (${m_{destroyed}}$) are set aside for the repair phase, while the positions of the remaining elements in ${S_{destroyed}}$ remain unchanged from those in ${S_{current}}$.

Five destroy strategies are developed in this study, which are introduced as follows:

*Operation Random Removal (ORR)*: The ORR operator randomly removes some elements from the current solution.

*Job Random Removal (JRR)*: The JRR operator randomly removes all operations corresponding to a job from the current solution.

*Critical Path Removal (CPR)*: The CPR operator deletes the operations on the critical path.

*Operation with Maximum Processing Time Removal (OMPTR)*: When there are multiple processing machines available for an operation and the machine currently selected for the operation has the greatest processing time, the operation is deleted by the OMPTR operator.

*Rework Job Removal (RJR)*: Remove all operations associated with the reworked job. Only used in the rescheduling stage.

#### Repair operators

The repair (insertion) operation reconstructs the destroyed solution ${s_{destroyed}}$ to generate a new solution ${s_{new}}$. Four tailored repair operators are developed as follows.

*Operation Random Insertion (ORI)*: The ORI operator randomly selects an operation from the removed elements ${m_{destroyed}}$ to insert into the destroyed solution ${s_{destroyed}}$. This operator can be utilized in pairs with the ORR, JRR, RJR and OMPTR operators.

*Job New Process Insertion (JNPI)*: The JNPI operator re-generates the process sequence of a job and inserts it into the destroyed solution. It can be utilized in pairs with the JRR and RJR operator.

*Critical Path Insertion (CPI)*: The CPI operator reconstructs the operations which are destroyed by the CPR operator.

*Operation with Minimum Processing Time Insertion (OMPTI)*: The OMPTI operator selects the machine with the smallest processing time for the removed elements ${m_{destroyed}}$, and inserts the corresponding operation into the destroyed solution ${s_{destroyed}}$. This operator can be utilized in pairs with the ORR, JRR, RJR and OMPTR operators.

All repair operators exhibit polynomial time complexity relative to problem size, which ensures the computational efficiency required for real-time rescheduling. Specifically, ORI and OMPTI execute in $O\left( {n^{\prime} \cdot m^{\prime}} \right)$ time, JNPI operates in $O\left( {n_{i}^{\prime } \cdot m^{\prime}} \right)$, and CPI completes in $O\left( {L \cdot m^{\prime}} \right)$, where $n^{\prime}$, $n_{i}^{\prime }$, *L*, and $m^{\prime}$ denote the total operations, job-specific operations, critical path length, and candidate machines, respectively.

#### Acceptance criteria

At each search step, a new solution ${S_{new}}$ is generated by applying problem-specific destroy and repair operators to the current solution ${S_{current}}$, guided by an RL-learned strategy. If ${S_{new}}$ dominates ${S_{current}}$, the current solution is replaced by the new solution. Otherwise, if the new solution fails to dominate the current one, it is accepted with a certain probability given by ${e^{ - \left( {c\left( {{S_{new}}} \right) - c\left( {{S_{current}}} \right)} \right)/T}}$. Here, *T* is the temperature starting from the initial temperature ${T_{start}}$, and it is updated according to: $T \leftarrow \alpha \cdot T$, where $\alpha$ is the cooling rate, and $0<\alpha <1$. Since this study addresses a multi-objective optimization problem, solution quality cannot be assessed by direct comparison of objective values. Instead, dominance is first evaluated. If neither solution dominates the other, crowding distance (i.e., $c\left( {{S_{new}}} \right)$ and $c\left( {{S_{current}}} \right)$) is used to differentiate them^13^.

### Details of RL-guided ALNS

Conventional ALNS relies solely on the historical performance of operators, potentially causing biased decisions. To address this limitation, this study proposes an RL-guided strategy that explicitly captures and exploits the underlying relationships within the current search environment. Specifically, the operator selection in ALNS is formulated as a Markov Decision Process (MDP), and RL is employed to adaptively select both destroy and repair operators.

#### State space

The state space provides a precise characterization of workshop environment and forms the foundation for decision-making in the RL-based scheduler. At scheduling time *t*, the state space ${S_t}$​ incorporates the attributes of all jobs $J{_t}$ and the status of all available machines ${M_t}$, formally expressed as ${S_t}=\left\{ {J{_t},~{M_t}} \right\}$. For the IPPS-SR problem under study, 18 representative state features are extracted to quantitatively describe the production environment, with detailed definitions provided in Table 4. These 18 features are carefully designed to fully capture the dynamic rework scenario and three types of flexibility inherent in IPPS‑SR. Covering job status, rework history, process flexibility, machine workload, and production stability, all features are indispensable for enabling the RL agent to make adaptive decisions.

**Table 4:** State features of the production environment for IPPS-SR.

ID	Description
$J_{t}^{1}$	Current rework operations
$J_{t}^{2}$	Status of each operation (0: not started; 1: completed; 2: in processing)
$J_{t}^{3}$	Percentage of uncompleted operations for each job
$J_{t}^{4}$	Total number of rework operations for each job
$J_{t}^{5}$	Historical rework rate of completed operations (Number of reworked operations / Total completed operations)
$J_{t}^{6}$	Mean remaining processing time of unscheduled jobs
$J_{t}^{7}$	Completion ratio of all operations (Number of completed operations / Total number of operations)
$J_{t}^{8}$	Number of feasible alternative routes for remaining operations of each job
$J_{t}^{9}$	Average number of substitutable machines available for unscheduled jobs
$M_{t}^{1}$	Machine processing quality level (Number of reworked operations on machine / Total operations processed on machine)
$M_{t}^{2}$	Number of unscheduled operations assigned to each machine
$M_{t}^{3}$	Workload for unscheduled operations to each machine
$M_{t}^{4}$	Number of machines currently blocked by rework operations
$M_{t}^{5}$	Mean machine utilization rate
$M_{t}^{6}$	Mean number of alternative machines for all unscheduled jobs
$M_{t}^{7}$	Mean utilization of machines
$M_{t}^{8}$	Maximum completion time of all machines
$M_{t}^{9}$	Machine assignment deviation rate from the original schedule

#### Action space

At each iteration of the MORL-ALNS algorithm, the agent’s action involves selecting a combination of a destroy (${\Omega ^d}$) and a repair (${\Omega ^r}$) operator from a predefined set (${\boldsymbol{\varOmega}^d}$ and ${\boldsymbol{\varOmega}^r}$). Guided by the policy ${\pi _\theta }$, the agent makes this selection based on the current state ${s_t}$. The selected operators are then sequentially applied to the current solution to generate a new candidate solution. Consequently, the policy function ${\pi _\theta }$ is refined as $\pi (\left( {{\Omega ^d},{\Omega ^r}} \right)|s,\theta )$.

#### Reward function

Designing an effective reward function (${R_t}$) is a fundamental challenge in RL, as it directly governs agent behavior. To this end, we adopt the reward function inspired by Kallestad et al.^56,57^ to evaluate the performance of operator combinations within our MORL-ALNS algorithm. The reward function evaluates operator combinations according to the quality of the resulting solutions, which can be summarized as follows:

14 $${R_t}=\left\{ {\begin{array}{*{20}{l}} {{\omega _1}}&{{\text{If }}{S_{new}}~{\text{can dominate all solutions in EP}}} \\ {{\omega _2}}&{{\text{If }}{S_{new}}~{\text{can dominate }}~{S_{current}}} \\ {{\omega _3}}&{{\text{If }}{S_{new}}~{\text{cannot dominate}}~{S_{current}},{\text{ but accepted}}} \\ {{\omega _4}}&{{\text{If }}{S_{new}}~{\text{is rejected}}} \end{array}} \right.$$

Here, ${\omega _1}>{\omega _2}>{\omega _3}>{\omega _4} \geqslant 0$. In this study, ${\omega _1}$, ${\omega _2}$, ${\omega _3}$ and ${\omega _4}$ are 10, 6, 3 and 0, respectively. EP represents the external Pareto archive. ${S_{current}}$ is the current solution, and ${S_{new}}$ denotes the new solution generated from ${S_{current}}$. This reward function evaluates the destroy and repair operators and improves the search process in the MORL-ALNS framework.

#### Training using proximal policy optimization

To enable the ALNS algorithm to intelligently and adaptively select destroy and repair operators in response to stochastic rework disturbances, a robust and efficient operator selection strategy ${\pi ^*}$ must be learned beforehand. This capability is crucial for achieving efficient search and intelligent trade-off balancing in the multi-objective IPPS-SR problem. This study employs the Proximal Policy Optimization (PPO) to optimize the parameter $\theta$ and refine the policy $\pi$. The training steps are detailed in Algorithm 1.

**Figure:**
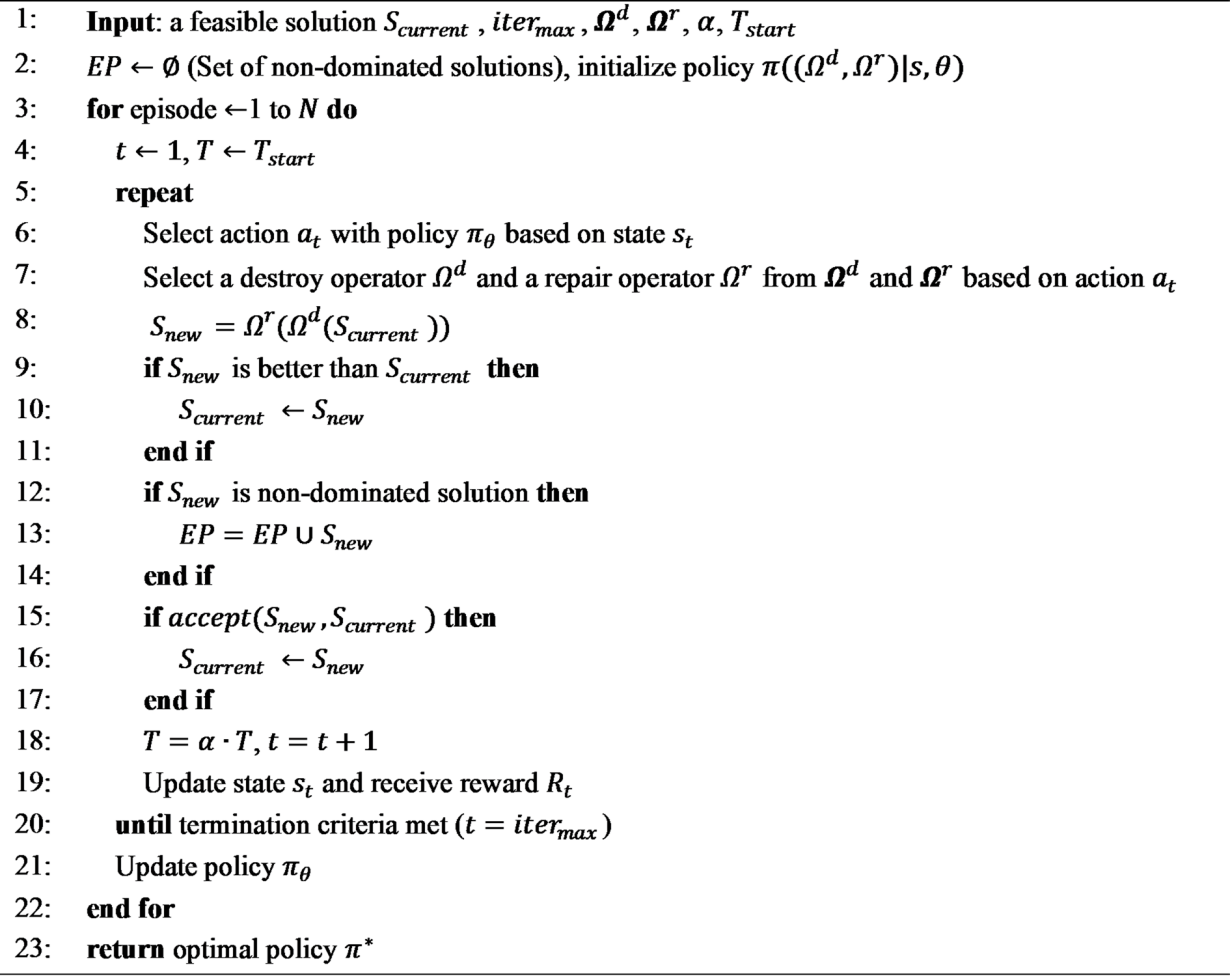
**Algorithm 1**: Pseudocode for training the optimal policy.

## Numerical experiments and discussion

As the right-shift strategy exerts no influence on the original scheduling results, we merely need to validate the effectiveness and superiority of the complete rescheduling algorithm MORL-ALNS. Accordingly, three sets of numerical experiments are conducted: (1) validating the algorithm parameters; (2) verifying the efficacy of the ALNS and RL-based guidance strategy; and (3) comparing the performance of the proposed method with other widely used algorithms. All benchmark problems and experimental results are archived in “https://github.com/CodeAndResearchData/IPPS-Rework”.

### Experimental environment and performance metrics

All algorithms are implemented in Python on a personal computer equipped with a 2.30 GHz i5 Intel Core processor and 8 GB random access memory (RAM) under the Windows 10 operating system. All experiments are performed with 10 independent runs, and the average values are adopted as the final results to mitigate the impact of randomness. Additionally, the proposed right-shift strategy is incorporated into all competing algorithms to ensure a fair comparison.

A set of typical IPPS benchmark instances, proposed by Kim et al.^58^, is tailored for the experiments. These 24 benchmark instances are constructed with 18 jobs (each having various flexibility levels) and 15 machines. To ensure model generality and experimental consistency, rework events are modeled as stochastic occurrences with fixed probabilities derived from industrial practice. The corresponding rework operations are randomly predetermined based on the given rework probability $rp$. In the following sections, these instances will be referred to as K1-K24. The following widely used performance metrics are employed to evaluate the algorithms in this study.

1. Number of non-dominated solutions (*NNDS*).

*NNDS* illustrates the number of Pareto solutions obtained by an algorithm, with a larger value indicating better performance. This metric is widely adopted for evaluating multi-objective algorithms^1,59,60^.

(2) Inverted generational distance (*IGD*).

*IGD* can measure both the diversity and convergence of the Pareto front, is defined as:

15 $$IGD\left( {P,{P^*}} \right)=\frac{1}{{\left| {{P^*}} \right|}}\mathop \sum \limits_{{x \in {P^*}}} \mathop {\hbox{min} }\limits_{{y \in P}} d\left( {x,y} \right)$$

where ${P^*}$ represents the optimal Pareto front, and *P* represents the obtained Pareto solution set. $\left| {{P^*}} \right|$ is the size of ${P^*}$. $d\left( {x,y} \right)$ denotes the Euclidean distance between *x* and *y*. The smaller the *IGD* value is, the better the algorithm’s performance. In this paper, ${P^*}$ consists of all non-dominated solutions obtained by all the compared algorithms.

(3) Hypervolume (*HV*).

*HV* can also represent the distribution and convergence of the solution set. The calculation of *HV* can be described as follows.

16 $$HV\left( {P,~r} \right)=volume\left( {{U_{f \in P}}\left[ {{f_1},{r_1}\left] { \times \cdots \times } \right[{f_m},{r_p}} \right]} \right)$$

where *P* is a solution set obtained by the compared algorithms. $r=\left( {{r_1},{r_2}, \cdots ,{r_p}} \right)$ represents the reference point for all objectives. In this paper, $r=\left( {{r_1},{r_2}} \right)=\left( {1.0,1.0} \right)$ has been selected as the reference point. The larger the *HV* value is, the better the algorithm’s performance.

### Effectiveness of ALNS operators

To validate the effectiveness of both the object-oriented encoding scheme and the five destroy operators and four repair operators (ALNS-OOE) proposed in this study for solving the IPPS-SR problem, a preliminary verification experiment is conducted on the static Kim benchmark instances^58^, with makespan as the optimization objective. Since many existing methods have been validated on these benchmark instances, they provide a reliable basis for comparison, enabling a direct demonstration of the effectiveness of the proposed operators. The comparison results with SEA^58^, IGA^61^, E-ACO^62^, GAVAS^63^, and SMGAVNS^1^ are presented in Table 5.

Table 5 presents the values of makespan, *RPD*, and computational time for all comparison algorithms. Column 1 lists the problem name. Column 2 and 3 report the number of jobs and the lower bound (LB) for each problem, respectively. Column 4 through Column 9 display the makespan values obtained by each comparison algorithm. Column 10 presents the *RPD* values between the results produced by ALNS-OOE (with the operators proposed in this study) and LB. Column 11 shows the computational time (CPU time, in seconds) used by ALNS-OOE.

As shown in Table 5, ALNS-OOE achieves the LB for 10 out of the 24 benchmark problems. Among all tested instances, the maximum *RPD* value relative to the LB is 0.25. For the makespan minimization objective, ALNS-OOE clearly outperforms SEA and IGA, while exhibiting performance comparable to E-ACO, GAVNS, and SMGAVNS. Furthermore, the maximum CPU time required is 225 s, which falls within an acceptable range. These results demonstrate that ALNS-OOE is an effective method for solving IPPS problems with the objective of makespan minimization. Although the optimization objective differs from the multi-objective functions considered in this study, namely minimizing makespan and operation and machine selection deviation, the validity of the proposed operators for solving IPPS-SR problems can be indirectly demonstrated through comparison with other algorithms.

**Table 5:** The value of makespan, *RPD*, and computational time of comparison algorithms.

Problem	*n*	LB	SEA	IGA	E-ACO	GAVNS	SMGAVNS	ALNS-OOE	RPD	CPU time (s)
K1	6	427	428	**427**	**427**	**427**	**427**	**427**	0.000	4.04
K2	6	343	**343**	**343**	**343**	**343**	**343**	**343**	0.000	3.94
K3	6	344	347	**344**	**344**	**344**	**344**	**344**	0.000	52.51
K4	6	306	**306**	**306**	**306**	**306**	**306**	**306**	0.000	9.42
K5	6	304	319	**304**	318	318	316	318	0.046	42.08
K6	6	427	438	**427**	**427**	**427**	**427**	**427**	0.000	37.67
K7	6	372	**372**	**372**	**372**	**372**	**372**	**372**	0.000	3.60
K8	6	342	343	**342**	343	343	351	343	0.003	76.39
K9	6	427	428	**427**	**427**	**427**	**427**	**427**	0.000	3.97
K10	9	427	443	**427**	**427**	**427**	**427**	**427**	0.000	101.88
K11	9	344	369	368	348	349	350	348	0.012	110.54
K12	9	306	328	312	322	319	317	319	0.042	129.89
K13	9	427	452	429	**427**	**427**	**427**	**427**	0.000	122.81
K14	9	372	381	386	373	379	393	379	0.019	73.88
K15	9	427	434	**427**	**427**	**427**	**427**	**427**	0.000	18.96
K16	12	427	454	433	429	**427**	437	429	0.005	107.56
K17	12	344	431	415	377	362	414	377	0.096	73.38
K18	12	306	379	364	357	349	358	349	0.141	78.47
K19	12	427	490	450	431	**427**	462	462	0.082	112.98
K20	12	372	447	429	386	383	419	419	0.126	86.79
K21	12	427	477	433	428	**427**	**427**	428	0.002	94.94
K22	15	427	534	491	444	433	476	476	0.115	50.32
K23	15	372	498	465	413	388	440	465	0.250	105.78
K24	18	427	587	532	460	446	493	504	0.180	225.00

### Parameter setting

To systematically calibrate the parameters of the proposed MORL-ALNS, Taguchi’s design of experiment (DOE) is employed. Seven parameters, each with three levels, are considered. The levels of each parameter are as follows: maximum iteration times $IterMax=\left\{ {1000,1500,2000} \right\}$, starting temperature ${T_{start}}=\left\{ {80,90,100} \right\}$; cooling rate $\alpha =\left\{ {0.90,0.95,0.98} \right\}$, reward parameters ${\omega _1}=\left\{ {10,12,15} \right\}$, ${\omega _2}=\left\{ {4,5,6} \right\}$, ${\omega _3}=\left\{ {0,2,3} \right\}$, ${\omega _4}=\left\{ { -2,-1,0} \right\}$. To reduce the experimental cost while preserving balanced coverage of the parameter space, an orthogonal array ${L_{27}}\left( {{3^7}} \right)$ is adopted. For each combination, the MORL-ALNS algorithm is independently run ten times on the IPPS-SR benchmark instances to account for stochastic variations. The average hypervolume (HV) value, denoted as the average response value (ARV), is calculated as the response variable, which reflects the overall solution quality in terms of both convergence and diversity.

The response values and significance rankings are presented in Table 6. The delta value for each parameter is derived from the range of its average ARV across the three levels, indicating the magnitude of its effect on algorithm performance. A larger delta implies greater influence. As shown in Table 6, $IterMax$ exhibits the largest delta (0.2491), ranking first in significance. This indicates that the search depth plays a dominant role in solution quality, as a higher maximum iteration allows the algorithm to explore the solution space more thoroughly. The cooling rate $\alpha$ and starting temperature ${T_{start}}$ ​rank second and third, respectively, suggesting that the annealing schedule substantially affects the balance between exploration and exploitation. Among the reward parameters, ${\omega _2}$ and ${\omega _3}$ show moderate effects, while ${\omega _1}$ and ${\omega _4}$​ have the least impact, with delta values below 0.05.

**Table 6:** Response value and significance rank of each parameter.

Level	$IterMax$	${T_{start}}$	$\alpha$	${\omega _1}$	${\omega _2}$	${\omega _3}$	${\omega _4}$
1	0.4978	0.5498	0.5104	0.6556	0.5526	0.5961	0.6041
2	0.6369	0.6256	0.6462	0.6167	0.6372	0.6154	0.6294
3	0.7469	0.7062	0.7249	0.6093	0.6918	0.67	0.648
Delta	0.2491	0.1564	0.2144	0.0462	0.1392	0.0739	0.0439
Rank	1	3	2	6	4	5	7

**Fig. 9:**
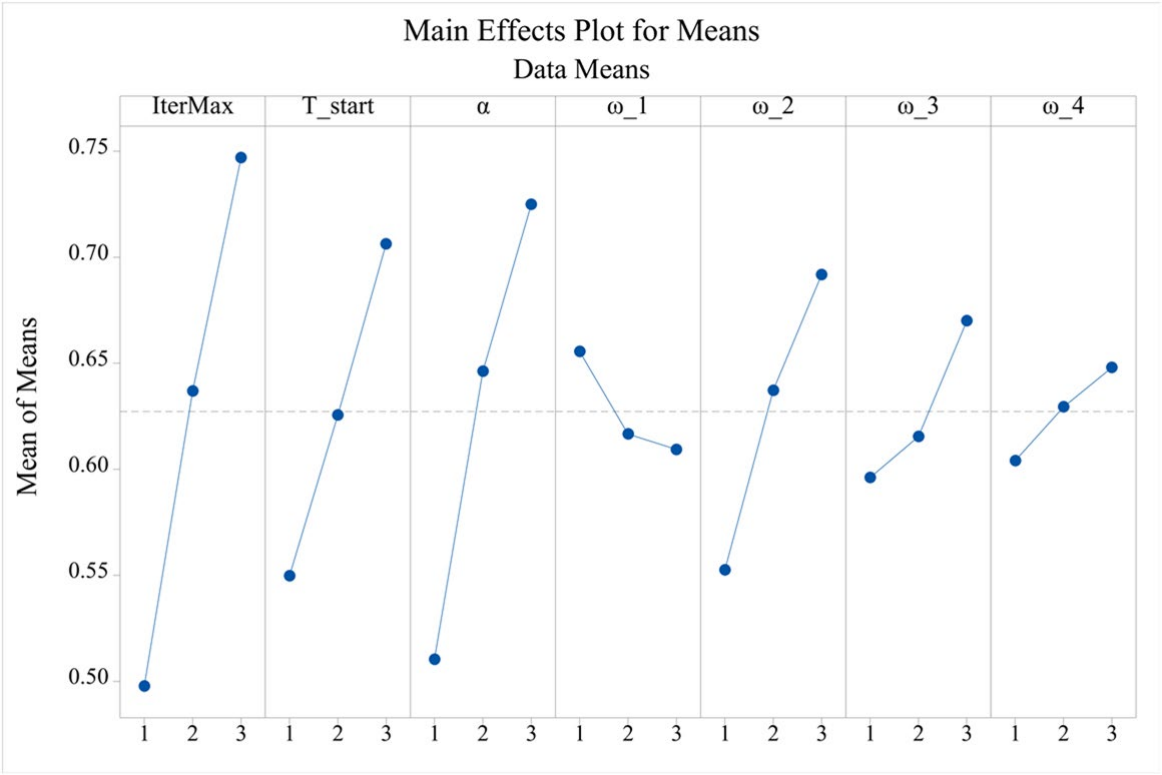
Factor trend levels of key parameters of MORL-ALNS.

Based on the level-wise trends shown in Fig. 9, the optimal parameter combination is identified as $IterMax=2000$, $\alpha =0.98$, ${T_{start}}=100$, ${\omega _1}=10$, ${\omega _2}=6$, ${\omega _3}=3$, ${\omega _4}=0$. This configuration achieves the highest ARV, demonstrating that a longer search process, a slower cooling schedule, and appropriately weighted reward signals collectively enhance the algorithm’s capability to approximate the Pareto front in the IPPS-SR problem.

### Effectiveness of the RL-guided operator selection method

To validate the effectiveness of the RL-guided mechanism in MORL-ALNS, experiments were conducted on 24 modified test instances under three rework probability scenarios (0.01, 0.02, and 0.03), with the *NNDS* adopted as the core evaluation metric. Table 7 compares the *NNDS* obtained by MORL-ALNS and ALNS methods at three rework probabilities. The first column displays the problem name. The total *NNDS* obtained by the two strategies are displayed in Columns 2, 5 and 8.

The results in Table 7 indicate that MORL-ALNS consistently generates a significantly higher number of *NNDS* than ALNS. Notably, in several cases (e.g., K8 at $rp=0.02$, K10 at $rp=0.01$), the ALNS failed to contribute any solutions (yielding zero), whereas MORL-ALNS consistently produced a substantial number of high-quality solutions. This demonstrates that the RL-guided mechanism substantially enhances solution space exploration capability and solution set quality.

**Table 7:** The *NNDS* obtained by different strategies at three rework probabilities.

Problem	$rp=0.01$			$rp=0.02$			$rp=0.03$		
	Total	ALNS	MORL-ALNS	Total	ALNS	MORL-ALNS	Total	ALNS	MORL-ALNS
K1	13	4	9	10	1	9	8	1	7
K2	20	5	15	15	3	12	12	4	8
K3	28	5	23	31	8	23	31	9	22
K4	19	4	15	13	5	8	29	10	19
K5	17	4	13	25	3	22	22	5	17
K6	16	5	11	28	9	19	13	4	9
K7	32	8	24	10	3	7	13	3	10
K8	14	3	11	14	0	14	12	1	11
K9	9	2	7	29	10	19	19	5	14
K10	14	0	14	24	3	21	18	1	17
K11	21	3	18	13	1	12	13	4	9
K12	23	4	19	21	4	17	22	5	17
K13	21	1	20	25	4	21	23	5	18
K14	16	4	12	14	0	14	24	0	24
K15	20	2	18	9	4	5	14	4	10
K16	19	5	14	17	4	13	16	3	13
K17	21	5	16	27	3	24	25	0	25
K18	7	1	6	15	5	10	24	3	21
K19	11	0	11	22	1	21	19	3	16
K20	11	4	7	21	1	20	22	5	17
K21	24	3	21	6	0	6	12	4	8
K22	15	3	12	9	3	6	15	5	10
K23	25	2	23	18	3	15	5	0	5
K24	10	1	9	12	0	12	25	3	22

**Fig. 10:**
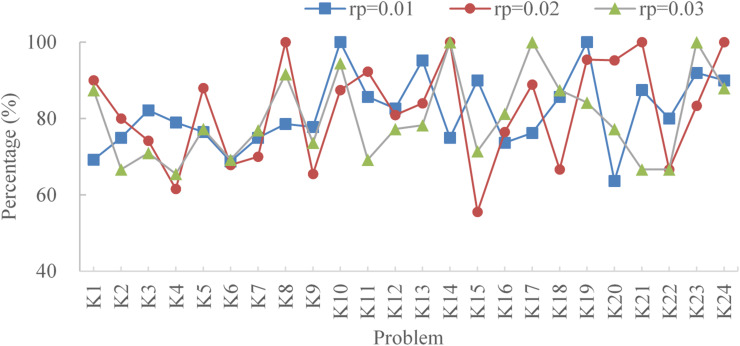
The percentage of *NNDS* obtained by MORL-ALNS under different rework probabilities.

Figure 10 displays the percentage of the *NNDS* obtained by MORL-ALNS relative to the total number under different rework probabilities. The percentage is calculated using Eq. (17).

17 $$Percentage=\frac{{NND{S_{MORL - ALNS}}}}{{NND{S_{total}}}} \times 100\%$$

$NND{S_{MORL - ALNS}}$ represents the number of non-dominated solutions obtained by MORL-ALNS, and $NND{S_{total}}$ is the total number of non-dominated solutions obtained by two strategies combined.

The visualization and corresponding numerical data in Fig. 10 show that MORL-ALNS maintains a high and stable contribution ratio: at $rp=0.01$, the percentage ranges from 63.64% (K20) to 100% (K10, K19). Specifically, 20 instances exceeding 75% and an average of 81.6%; at $rp=0.02$, the ratio varies between 55.56% (K15) and 100% (K8, K14, K21, K24), with 16 instances above 75% and an average of 82.1%; at $rp=0.03$, the percentage spans 66.67% (K21, K22) to 100% (K14, K17, K23), with 15 instances surpassing 75% and an average of 80.1%. To assess whether the observed superiority of MORL-ALNS over the baseline ALNS is statistically significant, we conduct the Wilcoxon signed-rank test on the NNDS values obtained from the 24 instances under each rework probability scenario. The results show that the improvements achieved by MORL-ALNS are statistically significant at the 0.05 significance level for all three rework probability settings (all *p*-value<0.05). These findings confirm that the RL-guided operator selection consistently outperforms the traditional ALNS across a wide range of uncertainty conditions.

The RL-guided mechanism in MORL-ALNS effectively addresses two key challenges in real-world production under rework uncertainty: generating a diverse set of trade-off solutions for multi-objective decision-making and maintaining robust performance across fluctuating uncertainty levels. Its consistently higher *NNDS* contribution across all instances, including challenging cases where the baseline ALNS yields zero, demonstrates superior search capability and reliability, which are critical for avoiding suboptimal resource allocation in industrial applications. Overall, these results confirm that the RL-based guiding strategy not only dominates solution sets in most scenarios, but also exhibits robust stability and low sensitivity to environmental uncertainties.

### The performance comparison with other algorithms

Three advanced algorithms are utilized to conduct a comparison in order to further evaluate the effectiveness of the MORL-ALNS. These include: non-dominated sorting genetic algorithm II (NSGA-II)^1^, multi-objective evolutionary algorithm based on decomposition (MOEA/D)^64^, and multi-objective Jaya (MOJaya)^65^. The reasons for choosing these three algorithms for comparison are as follows: (1) NSGA-II and MOEA/D are two widely used multi-objective optimization algorithms across various studies, and (2) MOJaya is currently a widely studied algorithm. To ensure the fairness of the comparison, each problem is independently run ten times. And parameter configurations of comparison algorithms are listed in Table 8. It is worth noting that the comparative experiments were conducted under all three rework probability settings ($rp=0.01,~0.02,~{\mathrm{and}}~0.03$) considered in this study, as the rework probability is a key factor influencing the dynamic scheduling environment. The results consistently show that MORL-ALNS outperforms the other competitive algorithms across all three probability levels. Due to space limitations, we present the results for $rp=0.02$ as a representative case, which represents a medium level of uncertainty.

**Table 8:** Parameter configurations of comparison algorithms.

Algorithm	Parameter settings
NSGA-II	population size = 100; crossover probability = 0.9; mutation probability = 0.4; maximum iteration times = 2000
MOEA/D	population size = 100; neighbor size = 5; Tchebycheff decomposition method; mutation probability = 0.2; maximum iteration times = 2000
MOJaya	population size = 100; mutation probability = 0.4; population reset probability = 0.1; maximum iteration times = 2000
MORL-ALNS	maximum iteration times ($IterMax$) = 2000; starting temperature (${T_{start}}$) = 100; cooling rate ($\alpha$) = 0.98; reward parameters ${\omega _1}=10$, ${\omega _2}=6$, ${\omega _3}=3$, ${\omega _4}=0$

Two performance metrics, namely IGD and HV, are adopted to evaluate the effectiveness of the comparison algorithms. The experimental results, including the mean metric values and standard deviation values of IGD and HV, are presented in Tables 9 and 10, respectively. The best mean metric value for each problem is highlighted in boldface.

**Fig. 11:**
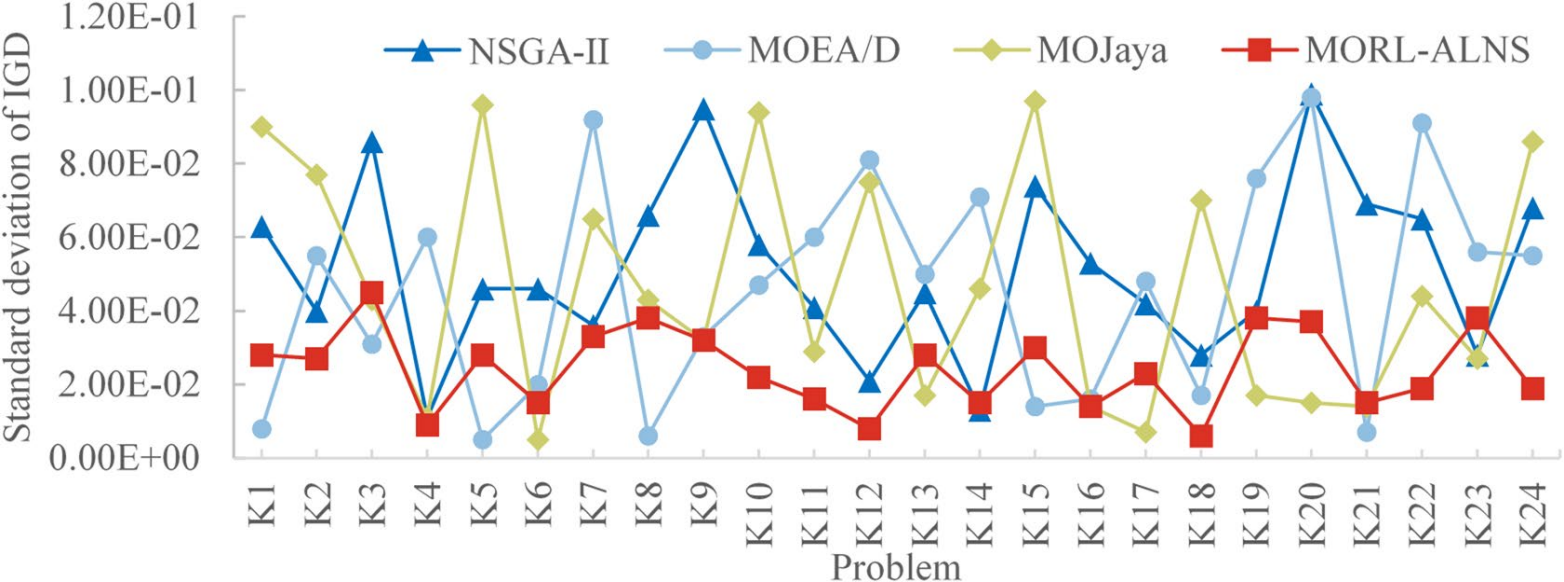
The standard deviation of IGD for comparison algorithms.

As shown in Table 9, for all test problems except K23, the IGD value of MORL-ALNS is lower than those of all other comparative algorithms. Although ALNS achieves a slightly better IGD value than MORL-ALNS on instance K23, the difference between them is only 1.82%. Further, Fig. 11 displays the standard deviation of IGD for all comparison algorithms. It can be seen that MORL-ALNS outperforms the other algorithms on 12 out of 24 problems. Regarding the other 12 problems, the standard deviation values of MORL-ALNS are comparable to those of the other algorithms. Therefore, it can be concluded that MORL-ALNS outperforms other algorithms, as reflected in the superior diversity and convergence of its obtained Pareto frontier solutions.

**Table 9:** Mean value and standard deviation of IGD obtained by four comparison algorithms.

Problem	NSGA-II		MOEA/D		MOJaya		MORL-ALNS	
	mean	std	mean	std	mean	std	mean	std
K1	0.175	0.063	0.211	0.008	0.215	0.090	**0.012**	0.028
K2	0.170	0.040	0.311	0.055	0.278	0.077	**0.090**	0.027
K3	0.232	0.086	0.216	0.031	0.173	0.043	**0.093**	0.045
K4	0.283	0.011	0.341	0.060	0.327	0.011	**0.280**	0.009
K5	0.162	0.046	0.168	0.005	0.224	0.096	**0.153**	0.028
K6	0.132	0.046	0.126	0.020	0.206	0.005	**0.037**	0.015
K7	0.192	0.036	0.242	0.092	0.097	0.065	**0.039**	0.033
K8	0.187	0.066	0.255	0.006	0.310	0.043	**0.086**	0.038
K9	0.137	0.095	0.230	0.033	0.286	0.032	**0.093**	0.032
K10	0.230	0.058	0.233	0.047	0.277	0.094	**0.033**	0.022
K11	0.331	0.041	0.235	0.060	0.303	0.029	**0.075**	0.016
K12	0.151	0.021	0.072	0.081	0.165	0.075	**0.071**	0.008
K13	0.337	0.045	0.372	0.050	0.318	0.017	**0.073**	0.028
K14	0.180	0.013	0.169	0.071	0.137	0.046	**0.126**	0.015
K15	0.165	0.074	0.126	0.014	0.170	0.097	**0.047**	0.030
K16	0.317	0.053	0.157	0.016	0.269	0.014	**0.138**	0.014
K17	0.263	0.042	0.334	0.048	0.214	0.007	**0.085**	0.023
K18	0.093	0.028	0.212	0.017	0.169	0.070	**0.019**	0.006
K19	0.309	0.040	0.232	0.076	0.392	0.017	**0.187**	0.038
K20	0.238	0.099	0.200	0.098	0.348	0.015	**0.074**	0.037
K21	0.207	0.069	0.098	0.007	0.247	0.014	**0.039**	0.015
K22	0.087	0.065	0.038	0.091	0.150	0.044	**0.036**	0.019
K23	**0.162**	0.028	0.237	0.056	0.239	0.027	0.165	0.038
K24	0.337	0.068	0.223	0.055	0.195	0.086	**0.157**	0.019
Mean Rank	2.79		2.88		3.29		**1.04**	

**Table 10:** Mean value and standard deviation of HV obtained by four comparison algorithms.

Problem	NSGA-II		MOEA/D		MOJaya		MORL-ALNS	
	mean	std	mean	std	mean	std	mean	std
K1	0.633	0.051	0.720	0.018	0.520	0.009	**0.721**	0.015
K2	0.779	0.059	0.821	0.032	0.240	0.090	**0.838**	0.032
K3	0.176	0.070	0.058	0.028	0.193	0.083	**0.709**	0.028
K4	0.749	0.034	0.761	0.091	0.739	0.088	**0.778**	0.026
K5	0.143	0.095	0.456	0.026	0.435	0.026	**0.505**	0.009
K6	0.760	0.063	0.499	0.041	0.284	0.005	**0.886**	0.030
K7	0.003	0.018	0.280	0.095	0.259	0.037	**0.363**	0.033
K8	0.746	0.061	0.826	0.053	0.928	0.069	**0.931**	0.035
K9	0.020	0.094	0.330	0.072	0.061	0.089	**0.968**	0.020
K10	0.260	0.086	0.321	0.038	0.450	0.039	**0.970**	0.052
K11	0.244	0.041	0.006	0.050	0.077	0.045	**0.821**	0.011
K12	**0.175**	0.080	0.114	0.063	0.164	0.061	0.167	0.054
K13	0.170	0.012	0.194	0.025	0.120	0.096	**0.472**	0.020
K14	0.154	0.031	0.237	0.034	0.228	0.052	**0.927**	0.039
K15	0.165	0.020	0.064	0.067	0.144	0.036	**0.264**	0.031
K16	0.744	0.083	0.464	0.008	0.630	0.069	**0.836**	0.006
K17	0.142	0.061	0.019	0.021	0.076	0.020	**0.950**	0.027
K18	0.494	0.059	0.655	0.017	0.668	0.022	**0.735**	0.016
K19	0.209	0.099	0.146	0.051	0.169	0.081	**0.899**	0.017
K20	0.310	0.029	0.170	0.040	0.120	0.008	**0.633**	0.019
K21	0.030	0.027	0.056	0.074	0.089	0.098	**0.677**	0.017
K22	0.248	0.073	0.371	0.089	0.216	0.010	**0.890**	0.013
K23	0.172	0.066	**0.179**	0.050	0.135	0.046	0.177	0.039
K24	0.141	0.030	0.135	0.078	0.094	0.049	**0.201**	0.034
Mean Rank	2.08		2.17		1.83		**3.92**	

**Fig. 12:**
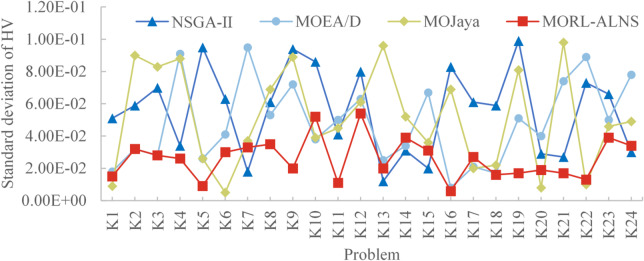
The standard deviation of HV for comparison algorithms.

Table 10and Fig. 12 present the results of four comparison algorithms with respect to the HV-metric. It can be observed that MORL-ALNS achieves larger mean HV values on 22 out of 24 problems, except for K12, and K23. And the difference in the mean HV between MORL-ALNS and the other algorithms for these two problems is slight. As for the standard deviation of HV, although MORL-ALNS has a greater value than others for 11 out of 24 problems, its mean value is much greater than that of the other three algorithms. Therefore, it can be concluded that the proposed MORL-ALNS achieves better convergence and diversity than NSGA-II, MOEA/D, and MOJaya.

To statistically validate the superior performance of the proposed MORL-ALNS, the Friedman test with the Nemenyi post-hoc procedure was conducted on the IGD and HV metrics across the 24 benchmark instances. As shown in Tables 9 and 10, MORL-ALNS achieves the best average rank for IGD (1.04) and also the best average rank for HV (3.92, where higher rank indicates better performance under this metric). The pairwise comparison results in Table 11 reveal that, for both IGD and HV, the performance differences between MORL-ALNS and each of the other three algorithms are statistically significant (adjusted *p*-values < 0.001). In contrast, no significant differences are observed among NSGA‑II, MOEA/D, and MOJaya (all adjusted *p*-values = 1.000). These statistical findings confirm that the proposed MORL-ALNS significantly outperforms the compared algorithms.

**Table 11:** Nemenyi test for pairwise comparisons among all methods (the level of significant $\alpha ~=~0.05$).

Metric	Algorithm pair	Unadjusted *p*-value	Adjusted *p*-value ($\alpha =0.05$)
IGD	MORL-ALNS vs. NSGA-II	0.000	0.000
	MORL-ALNS vs. MOEA/D	0.000	0.000
	MORL-ALNS vs. MOJaya	0.000	0.000
	NSGA-II vs. MOEA/D	0.823	1.000
	NSGA-II vs. MOJaya	0.180	1.000
	MOEA/D vs. MOJaya	0.264	1.000
HV	MORL-ALNS vs. NSGA-II	0.000	0.000
	MORL-ALNS vs. MOEA/D	0.000	0.000
	MORL-ALNS vs. MOJaya	0.000	0.000
	MOEA/D vs. NSGA-II	0.823	1.000
	MOEA/D vs. MOJaya	0.371	1.000
	NSGA-II vs. MOJaya	0.502	1.000

**Fig. 13:**
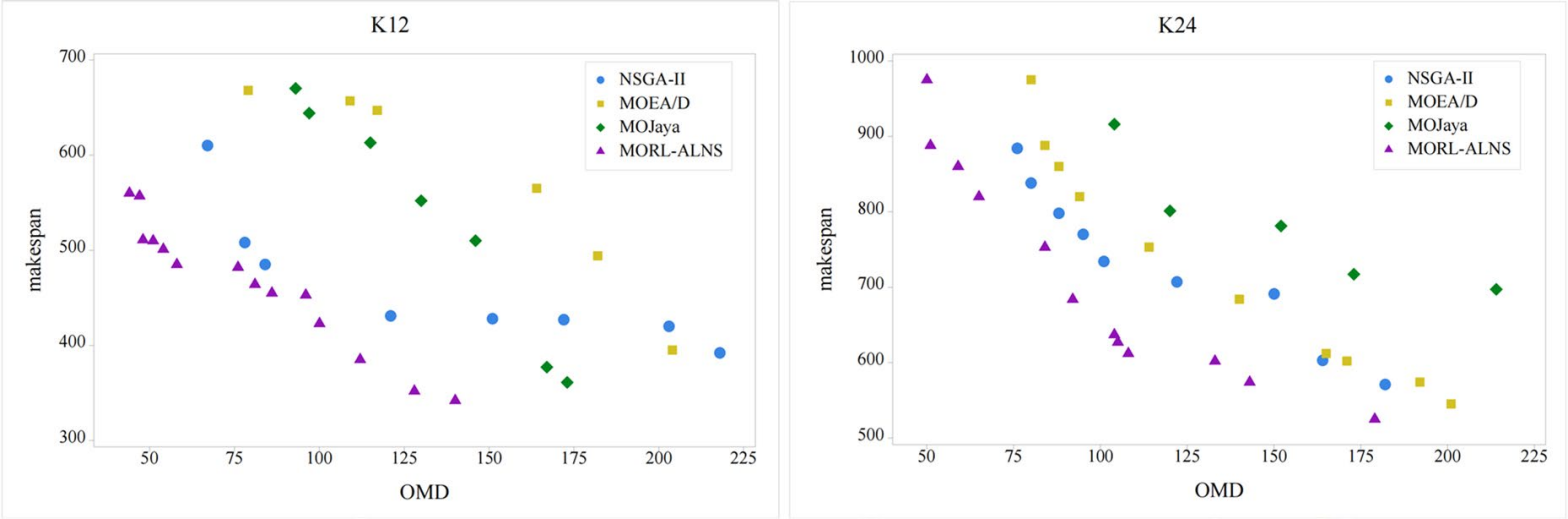
Pareto sets obtained by four algorithms on K12 and K24.

Furthermore, in order to provide an intuitive illustration of the trade-off between the two objectives, Fig. 13 depicts the Pareto fronts obtained by MORL-ALNS and the three comparison algorithms on two benchmark instances, K12 and K24, under the rework probability setting $rp=0.02$. The results also show that MORL-ALNS consistently achieves better convergence and diversity, producing a well-distributed set of non-dominated solutions that effectively balance makespan and schedule instability.

**Fig. 14:**
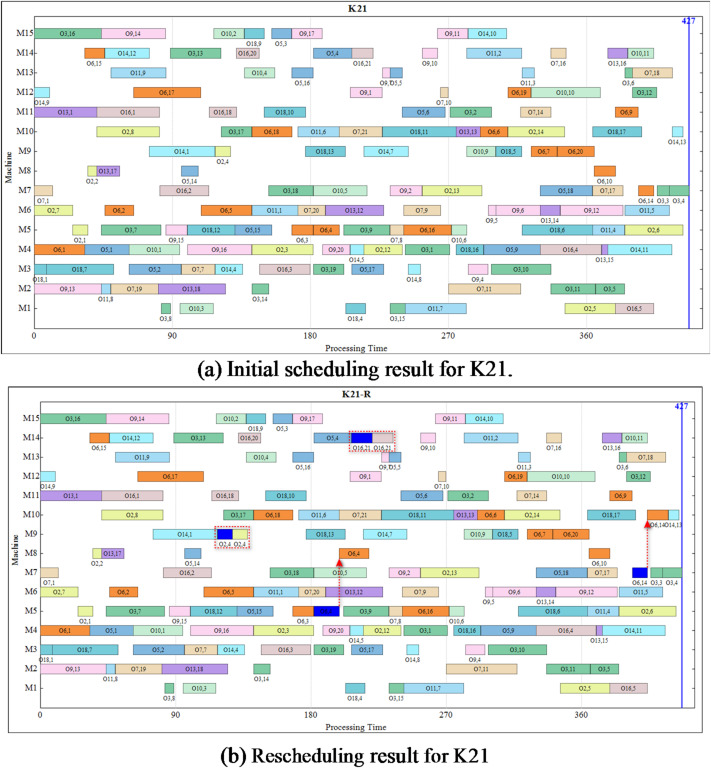
An example of scheduling results for K21. (a) Initial scheduling result for K21., (b) Rescheduling result for K21.

Figure 14 depicts an example of initial scheduling and rescheduling scheme for K21, revealing that ${O_{2,4}}$, ${O_{6,4}}$, ${O_{16,21}}$ and ${O_{6,14}}$ are rework operations. In accordance with the rescheduling strategy proposed in this paper, ${O_{2,4}}$ and ${O_{16,21}}$ are reproduced immediately on their original machines without necessitating a complete rescheduling. For ${O_{6,4}}$, operational flexibility is exploited by moving it from ${M_5}$ to ${M_8}$. This relocation increases its processing time from 17 to 20, but successfully prevents delays to other operations. In contrast, rework on operation ${O_{6,14}}$ necessitates a complete rescheduling. Reprocessing it on its original machine ${M_7}$ would delay operations ${O_{3,3}}$ and ${O_{3,4}}$, thereby increasing the makespan. Conversely, reassigning it to ${M_{10}}$ only delays ${O_{14,13}}$ and does not extend the makespan. As can be seen from Fig. 14, it can also be concluded that the proposed method is highly effective in addressing the IPPS-SR problem.

The performance analysis and comparison demonstrate that the proposed MORL-ALNS algorithm outperforms other existing well-known multi-objective optimization algorithms in resolving the IPPS-SR problem.

## Conclusions and future works

This study tackles the IPPS-SR problem by developing a multi-objective optimization model and introducing an event-driven hybrid rescheduling framework. The framework integrates a right-shift strategy with a RL-guided ALNS algorithm. During the complete rescheduling phase, MORL-ALNS dynamically adjusts the selection probabilities of five destroy operators and four repair operators through a RL mechanism, achieving an effective balance between search exploration and exploitation. Experimental results confirm that the proposed hybrid rescheduling approach outperforms comparison algorithms in terms of solution quality, convergence, and diversity, which validates its efficacy in solving the IPPS-SR problem.

In future research, the proposed model could be extended to incorporate more dynamic events, such as machine breakdown, new job arrival, and order cancellation. Furthermore, additional investigations into rescheduling policies can be conducted, and other objectives such as earliness, tardiness, and environmental factors can be considered in the problem formulation. The proposed approach will also be tested and verified on more datasets from real-world manufacturing scenarios to further validate its practicality and generalization ability. Moreover, future studies should further investigate the rationality and generalizability of the assumptions underlying the research objectives, including job–machine processing constraints, setup and transportation time aggregation, rework time definition, and relaxing these assumptions will help improve the model’s applicability and robustness in more realistic production scenarios.

## Data Availability

The datasets generated and analyzed during the current study are available at [https://github.com/CodeAndResearchData/IPPS-Rework](https:/github.com/CodeAndResearchData/IPPS-Rework) .
